# Star-PAP RNA Binding Landscape Reveals Novel Role of Star-PAP in mRNA Metabolism That Requires RBM10-RNA Association

**DOI:** 10.3390/ijms22189980

**Published:** 2021-09-15

**Authors:** Ganesh R. Koshre, Feba Shaji, Neeraja K. Mohanan, Nimmy Mohan, Jamshaid Ali, Rakesh S. Laishram

**Affiliations:** 1 Cardiovascular Diseases & Diabetes Biology, Rajiv Gandhi Centre for Biotechnology, Trivandrum 695014, India; ganeshram@rgcb.res.in (G.R.K.); febashaji@rgcb.res.in (F.S.); neerajakm@rgcb.res.in (N.K.M.); nimmym@rgcb.res.in (N.M.); 2 Manipal Academy of Higher Education, Manipal 576104, India; 3 Regional Centre for Biotechnology, Faridabad 121001, India; 4 Bioinformatics Facility, Rajiv Gandhi Centre for Biotechnology, Trivandrum 695585, India; jam@rgcb.res.in

**Keywords:** 3′-end processing, polyadenylation, Star-PAP, RBM10, HITS-CLIP, mRNA metabolism, RNA-turnover

## Abstract

Star-PAP is a non-canonical poly(A) polymerase that selects mRNA targets for polyadenylation. Yet, genome-wide direct Star-PAP targets or the mechanism of specific mRNA recognition is still vague. Here, we employ HITS-CLIP to map the cellular Star-PAP binding landscape and the mechanism of global Star-PAP mRNA association. We show a transcriptome-wide association of Star-PAP that is diminished on Star-PAP depletion. Consistent with its role in the 3′-UTR processing, we observed a high association of Star-PAP at the 3′-UTR region. Strikingly, there is an enrichment of Star-PAP at the coding region exons (CDS) in 42% of target mRNAs. We demonstrate that Star-PAP binding de-stabilises these mRNAs indicating a new role of Star-PAP in mRNA metabolism. Comparison with earlier microarray data reveals that while UTR-associated transcripts are down-regulated, CDS-associated mRNAs are largely up-regulated on Star-PAP depletion. Strikingly, the knockdown of a Star-PAP coregulator RBM10 resulted in a global loss of Star-PAP association on target mRNAs. Consistently, RBM10 depletion compromises 3′-end processing of a set of Star-PAP target mRNAs, while regulating stability/turnover of a different set of mRNAs. Our results establish a global profile of Star-PAP mRNA association and a novel role of Star-PAP in the mRNA metabolism that requires RBM10-mRNA association in the cell.

## 1. Introduction

Pre-mRNA 3′-end processing is an essential step in eukaryotic gene expression that involves two coupled steps-endonucleolytic cleavage followed by the addition of a poly(A) tail (polyadenylation) [1,2,3,4]. 3′-end processing is carried out by a cleavage and polyadenylation complex (CPA) that is associated with >85 protein components [3,5,6]. The CPA complex is comprised of subunits of cleavage and polyadenylation specificity factor (CPSF), cleavage stimulatory factor (CstF), cleavage factors Im (CFIm) and IIm (CFIIm), scaffolding protein symplekin, poly(A) polymerase (PAP), and poly(A) binding protein (PABPN1) as core components [3,4]. Cleavage and polyadenylation at the pre-mRNA 3′-end involve recognition of a poly(A) signal (PA-signal) by CPSF-30 and WDR33 subunits of CPSF complex [7,8,9,10]. CstF (CSTF2) interacts with the GU/U-rich downstream sequence (DSE) and cooperates with CPSF to assemble a stable CPA complex [11,12]. CPSF3 then cleaves pre-mRNA at the PA-site followed by PA-tail addition by a PAP on the upstream fragment, whereas the downstream fragment is rapidly degraded [13,14]. PABPN1 then binds and stabilises the PA-tail and controls PA-tail length [15,16,17]. Canonical PAPα/γ is the primary PAP for mRNA polyadenylation in the nucleus [18,19]. The discovery of a variant of PAP, Star-PAP, reveals the existence of alternative PAP for nuclear polyadenylation [1,20,21]. Star-PAP selectively polyadenylates mRNAs involved in oxidative stress, apoptosis, cancer and cardiac hypertrophy [21,22,23,24,25,26,27]. In addition to the polyadenylation activity, Star-PAP has a confirmed uridylation activity [21,28,29], and has been implicated in the regulation of miRNA biogenesis and stability [30,31].

Approximately, 70% of human genes have multiple PA-sites that are alternately used (alternative polyadenylation, APA) generating diversity of mRNA isoforms [32,33]. Recently, we showed a genome-wide mechanism of APA wherein canonical PAPα and γ, and Star-PAP selects distinct PA-sites on target mRNAs [18]. Star-PAP primarily selects the PA-distal site resulting in a predominant 3′-UTR shortening on Star-PAP knockdown [18]. Yet, the mechanism of how Star-PAP selects target PA-site and assembles specific CPA complex is unclear. We identified a Star-PAP recognition element on the target mRNA 3′-UTRs (*HMOX1*, *BIK* and *NQO1*) and demonstrated that canonical PAPα/γ is excluded from Star-PAP targets enabling selective polyadenylation of target mRNA [20,25,34]. Mass spectrometry analysis of Star-PAP associated proteins identified RNA binding motif 10 (RBM10) as a unique Star-PAP complex component absent in the canonical PAPα/γ complex [24]. RBM10 is an RNA binding protein that recognises homopolymers of G or U ribonucleotides in vitro [35,36,37,38]. A mutational defect in RBM10 was reported to cause an X-linked recessive disorder (TARP syndrome) and lung adenocarcinoma [39,40]. RBM10 has high homology with other RBMs such as RBM5 involved in apoptosis [41,42]. Studies have demonstrated the role of RBM10 in alternative splicing and 3′-end processing [24,42,43]. RBM10 associates with different spliceosome complexes, hnRNPs, and U2 snRNPs and controls alternative splicing of apoptosis-related genes *FAS* and *BclX* [43,44,45,46,47,48]. Genome-wide RNA-Seq demonstrated significant skipped exons upon depletion of RBM10 in HEK 293 cells [42]. We show a splicing independent function of RBM10 that regulates 3′-end processing of Star-PAP target mRNAs involved in hypertrophy gene program [24]. Yet the global role of RBM10 on Star-PAP 3′-UTR association is unclear.

In this study, we carried out a HITS-CLIP experiment to map the Star-PAP binding landscape and to define the mechanism of Star-PAP target 3′-end PA-site selection. We observed a genome-wide association of Star-PAP in all the chromosomes that was lost on Star-PAP depletion. Consistent with its role in the 3′-UTR processing, we observed a high association of Star-PAP at the 3′-UTR region. Using 3′-RACE assay and RNA immunoprecipitation, we confirmed that Star-PAP global 3′-UTR association regulates 3′-end processing of target mRNAs. Strikingly, there was an enrichment of Star-PAP at the coding region exons (CDS) in more than 40% of target mRNA that was independent of the Star-PAP 3′-end processing function. We demonstrated that Star-PAP binding regulates the half-life of these mRNA targets indicating a new role of Star-PAP in mRNA metabolism. To further define the mechanism of the Star-PAP RNA association, we depleted RBM10 and carried out the Star-PAP HITS-CLIP experiment. Strikingly, we observed global loss of Star-PAP association (over 70% target mRNAs) on target RNAs on RNAi mediated RBM10 depletion. This includes both mRNAs where Star-PAP was associated at the 3′-UTR and CDS regions. Consistently, RBM10 depletion compromises 3′-end processing of a set of Star-PAP target mRNAs, whereas it regulates the stability and turnover of a different set of Star-PAP target mRNAs. Together, our results established a new role of Star-PAP in mRNA metabolism that is controlled through RBM10-RNA association.

## 2. Results

### 2.1. Genome-Wide Star-PAP RNA Binding Landscape Reveals Star-PAP Direct mRNA Targets

Star-PAP is a variant PAP that controls the expression of mRNAs involved in multiple cellular functions. Genome-wide microarray analysis after Star-PAP knockdown demonstrated both up-regulated and down-regulated sets of mRNAs [21,49]. Yet, how many of such altered mRNAs are directly controlled by Star-PAP or the mechanism of Star-PAP target PA-site/3′-UTR selection is still undefined. To assess this, we performed HITS-CLIP sequencing after pull-down of Star-PAP in the presence and absence of RBM10 depletion with IgG as a reference control. HITS-CLIP of Star-PAP was further confirmed after Star-PAP depletion in a similar HITS-CLIP experiment.

**Figure 1:**
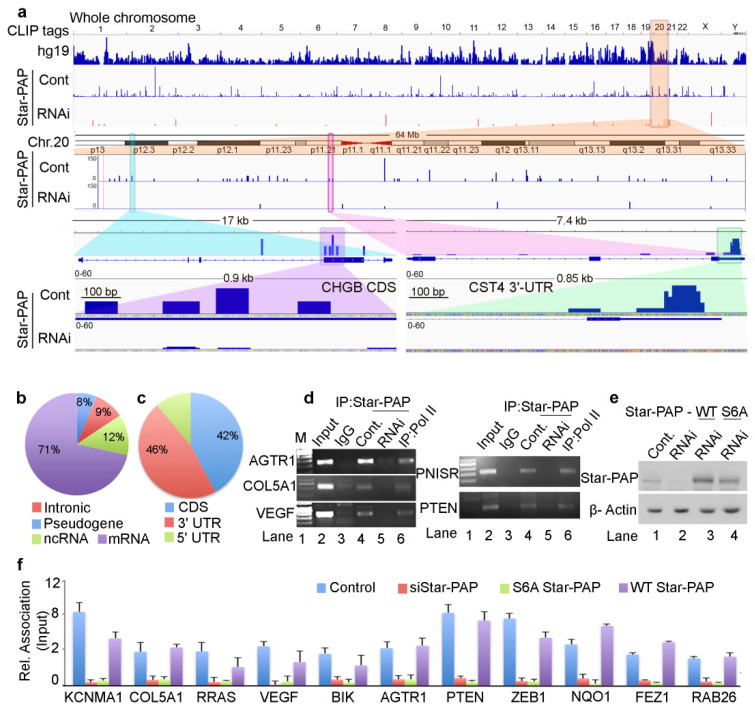
Star-PAP genome-wide binding landscape across the human genome shows a variety of mRNA targets. (**a**) HITS-CLIP signals of Star-PAP distribution across human genome showing whole chromosomal association of Star-PAP in control and siRNA Star-PAP depletion in HEK 293 cells. Star-PAP HITS-CLIP tag distribution in one example chromosome (chromosome 20) and the loss of the mapped tags on siStar-PAP is shown below. Similar distribution on 6 selected chromosomes is shown in Supplementary Figure S1a. The CLIP tags here and in the supplementary figures were counted after peak calling. Enlarged 17-kb region of *CHGB* mRNA and 7.4-kb *CST4* mRNA along with mapped cluster region in the *CHGB*-CDS and *CST4*-UTR regions is shown below. (**b**) Distribution Star-PAP associations among different RNA species (protein-coding (mRNA), non-coding RNAs (ncRNA), pseudo genes, and intronic RNA regions are indicated). (**c**) Distribution of Star-PAP associated nucleotide positions of mRNAs among coding exons (CDS), 3′-UTR and terminal exon, and the 5′-UTR regions. (**d**) RNA immunoprecipitation (RIP) analysis of Star-PAP in control and siRNA treated cells and the control Pol II on various Star-PAP targets as indicated. Input, 10% of the IP lysate. M, marker lane. (**e**) Western blot analysis of Star-PAP and control β-actin in control and Star-PAP depleted HEK 293 cells in the presence and absence of rescue with wild type and S6A mutant Star-PAP. (**f**) Quantitative RIP (qRIP) analysis of various Star-PAP target mRNAs detected in the HITS-CLIP experiment as indicated, showing the relative association of Star-PAP in control, Star-PAP knockdown and Star-PAP RNA binding mutant (S6A) Star-PAP expressed cells. (S6A Star-PAP carrying silent mutations that render siRNA ineffective was ectopically expressed in the cell after endogenous Star-PAP knockdown). Error bar represents SEM (standard error mean) of three independent experiments.

HITS-CLIP sequencing raw data of each Star-PAP sample had approximately 10 million reads. After filtering for quality reads, removing adapter sequences and identical reads from PCR amplification, we obtained ~8 million sequencing reads that were used for aligning to the reference genome. Further, parsing the alignment to obtain uniquely mapped reads, and a minimal mapping size of 18 nucleotides, we generated about 4.2 million distinct sequencing reads that uniquely mapped to the human reference genome (hg19). The association of identified reads was observed in all chromosomes with a varying number of mapped reads (Figure 1a). The highest mapped reads were observed on chromosomes 1, 8 and 19 and the lowest reads were observed on Chromosome Y (Supplementary Figures S1a and S2a). After peak calling and subtraction for reference IgG HITS-CLIP, we identified 420,000 read clusters corresponding to ~14,000 distinct transcripts in Star-PAP HITS-CLIP. To confirm the specificity of Star-PAP binding, we employed siRNA-mediated depletion of Star-PAP and a similar HITS-CLIP experiment was carried out. Interestingly, depletion of Star-PAP resulted in the loss of 65% of the mapped regions on the reference genome (Figure 1a, Supplementary Figure S2a). The loss of read clusters was observed in almost all chromosomes with varying degrees (Supplementary Figure S2a). Reduced maps on Star-PAP knockdown in some of the chromosomes (chromosome 2, 4, 7, 17, 13, 19, and 20) and percent reductions in respective chromosomes are shown in Figure 1a, Supplementary Figures S1a and S2a. To further assess the difference in the Star-PAP associated clusters between control and Star-PAP depletion on specific mRNAs, we enlarged a region of 17-kb at *CHGB* mRNA and a 7-kb region around *CST4* mRNA from chromosome 20 (Figure 1a). We also enlarged a 200-bp region around the peak cluster that shows Star-PAP associated nucleotide sequence on the mRNA (Supplementary Figure S2c). Together, these results reveal different binding regions of Star-PAP on different mRNAs that were lost on siStar-PAP depletion.

Mapping of the genetic regions of Star-PAP association showed Star-PAP binding sites were primarily associated with protein-coding RNAs (>70%) with less than 15% in the non-coding RNAs (miRNA, snRNA, lncRNA and snoRNA), and ~10% in the intronic RNAs (Figure 1b). For the analysis of Star-PAP specific target protein-coding mRNAs, we considered transcripts that had high read detection (>10-read tags per cluster) and those absent from the HITS-CLIP experiment after Star-PAP depletion. With this stringent condition, we obtained 4200 specific mRNAs directly associated with Star-PAP (Supplementary Table S1). Among the specific protein-coding mRNAs, significant Star-PAP association was detected at the CDS (exons) region, 3′-UTR and terminal exons, and in the 5′-UTR regions (Figure 1c). Overall, Star-PAP detection was high at the 3′-UTR regions (46%) consistent with its primary role in the 3′-UTR processing. Interestingly, the detection in the CDS region was equally high (42%) (Figure 1c) indicating a distinct role of Star-PAP in mRNA metabolism. Moreover, in many of the transcripts, Star-PAP was detected at both CDS and 3′-UTR regions. Three mRNAs with Star-PAP association at the 3′-UTR and CDS regions are shown in Figure 1a and Supplementary Figure S2c. Yet, how Star-PAP binding at the CDS region alters gene expression is not understood (detailed in the following sections). Consistent with earlier studies of the Star-PAP target mRNAs, our in silico analysis of nucleotide composition of these Star-PAP specific reads confirms a biased GC content over AU in the Star-PAP bound regions in both 3′-UTR and CDS regions (Supplementary Figure S1b). Analysis of consensus sequence motif of 12-mers using MEME-Chip software confirmed an -AUA- containing consensus motif at the target mRNAs (Supplementary Figure S2b). An enlarged region of the Star-PAP read cluster at the 3′-UTR of target mRNA also shows a similar motif with an -AUA- sequence (Supplementary Figure S2c). This is in line with our earlier in vitro footprinting data and in silico target analysis reinforcing the earlier role of Star-PAP in the 3′-end processing of target mRNAs [20,25].

### 2.2. Star-PAP Associated mRNA Targets Show Wide Roles of Star-PAP in Human Diseases and Signaling Pathways

To validate our HITS-CLIP experiments, we performed qualitative RNA immunoprecipitation (RIP) and quantitative RIP (qRIP) analysis of selected Star-PAP target mRNAs. We observed the association of Star-PAP with mRNAs including the earlier established targets (*AGTR1*, *COL5A1*, *VEGF*, *PTEN*, *PNISR*, *IFRD1*) by qualitative RIP analysis (Figure 1d). Star-PAP knockdown resulted in the loss of association. Control RNA Pol II was associated with all mRNAs tested (Figure 1d). Similarly, in the qRIP analysis as well, we observed the association of Star-PAP on mRNAs (*KCNMA1*, *COL5A1*, *RRAS*, *VEGF*, *BIK*, *AGTR1*, *PTEN*, *ZEB1*, *NQO1*, *FEZ1*, *RAB26*) that was lost on Star-PAP knockdown (Figure 1f). In addition, Star-PAP RNA binding mutant (S6A Star-PAP) [50] but not wild type Star-PAP also resulted in the loss of binding to target mRNAs (Figure 1f). Western analysis of siRNA Star-PAP depletion and rescue with wild type and S6A Star-PAP is shown in Figure 1e. To gain further insight into cellular functions of Star-PAP target mRNAs, we analysed Star-PAP bound mRNAs from HITS-CLIP sequencing for functional pathways in different human diseases and cellular signals (Supplementary Figure S1c–d). We observed enrichment of mRNAs involved in cancer, heart disease, metabolic diseases, immunity and infection among the Star-PAP bound targets (Supplementary Figure S1c). Among the signalling pathways, RTK-MAPK, PI3K-Akt, GPCR, Interleukin and Wnt signalling pathways were enriched among the target mRNAs (Supplementary Figure S1d) indicating a wide function of Star-PAP in the cell.

### 2.3. Star-PAP RNA Binding Can Both Down Regulate and Up-Regulate Target mRNA Expression

**Figure 2:**
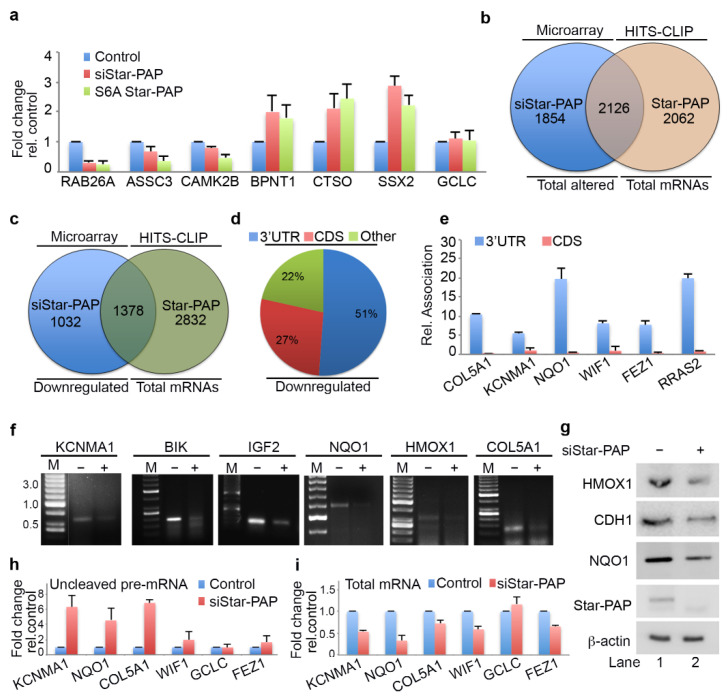
Star-PAP global mRNA association regulates the 3′-end processing of target mRNAs. (**a**) qRT-PCR analysis of select Star-PAP associated mRNAs from HITS-CLIP experiment with total RNA isolated from HEK 293 cells with control, Star-PAP, and with exogenous S6A Star-PAP expression after silencing of endogenous Star-PAP in HEK 293 cells, as in Figure 1e. The error bars indicate standard errors of the mean (SEM). (**b**) Venn diagram showing overlapped mRNA targets detected in Star-PAP HITS-CLIP and significantly altered (both down-regulated and up-regulated) mRNAs on Star-PAP depletion from earlier microarray analysis. (**c**) Venn diagram showing overlapped mRNA targets between down-regulated genes on Star-PAP depletion from earlier microarray analysis and mRNAs associated with Star-PAP detected in Star-PAP HITS-CLIP. (**d**) Distribution of Star-PAP associated nucleotide positions in the coding region and 3′-UTR region among overlapped down-regulated mRNAs on Star-PAP knockdown that is detected in Star-PAP HITS-CLIP experiment. (**e**) qRIP analysis showing the relative association of Star-PAP in the coding region and 3′-UTR region on select Star-PAP target mRNAs as indicated. Error bar represents SEM of three independent experiments. (**f**) 3′-RACE assay of various Star-PAP target mRNAs (*KCNMA1*, *IGF2*, *HMOX1*, *BIK*, *COL5A1*, *NQO1*) from total RNA isolated from HEK 293 cells after Star-PAP knockdown. The absence of siRNA (-siRNA) indicates that control-scrambled siRNA was used. (**g**) Western blot analysis of Star-PAP, target proteins, and control actin from control and Star-PAP knockdown HEK 293 cells. (**h**,**i**) Measurement of uncleaved pre-mRNA levels (**h**) expressed relative to total mRNA (**i**) after Star-PAP knockdown or control cells as indicated.

Interestingly, qRT-PCR analysis indicated the down-regulation of a number of Star-PAP-associated mRNAs on Star-PAP depletion consistent with Star-PAPs role in the 3′-end processing (Figure 2a, Supplementary Figure S2d). However, a number of mRNAs (*BPNT1*, *CTSO*, *SSX2*) that were bound by Star-PAP were up-regulated on Star-PAP knockdown (Figure 2a) suggesting a distinct function of Star-PAP in mRNA metabolism in addition to the 3′-end processing. These mRNAs were equally up-regulated in the presence of S6A Star-PAP RNA binding mutation. There was no effect of Star-PAP knockdown on the control non-target *GCLC* mRNA (Figure 2a). To gain further mechanistic insight into the role of Star-PAP in RNA metabolism, we compared mRNAs detected in our HITS-CLIP Star-PAP with that of earlier microarray analysis that showed altered expression on Star-PAP depletion (~1500 genes up-regulated and ~2400 genes down-regulated) [49]. Around 55% of the mRNAs whose expression was significantly altered on Star-PAP knockdown in microarray analysis were detected in our Star-PAP HITS-CLIP (Figure 2b) indicating that the expression of these set of mRNAs is directly regulated by Star-PAP-RNA association. Interestingly, there was a higher occurrence of down-regulated mRNAs than that of up-regulated mRNAs in the Star-PAP HITS-CLIP. There was ~60% of the down-regulated genes on Star-PAP depletion was detected in the Star-PAP HITS-CLIP (Figure 2c). Consistently, Star-PAP was primarily detected at the 3′-UTR and the terminal exon among these mRNAs (Figure 2d). Moreover, among the mRNA from the HITS-CLIP data where Star-PAP was detected at the 3′-UTR region, the majority were down-regulated on Star-PAP depletion in the microarray data (Supplementary Figure S2e). The Association of Star-PAP on a select mRNA at the 3′-UTR is shown in Supplementary Figure S2c. Together these results reveal that the Star-PAP association at the 3′-UTR region regulates the 3′-end processing of target mRNAs.

Further, qRIP analysis of 6 select mRNAs that were down-regulated on Star-PAP depletion (*COL5A1*, *KCNMA1*, *WIF1*, *NQO1*, *FEZ1*, *RRAS2*) demonstrated a biased association of Star-PAP at the 3′-UTR compared to the CDS regions (Figure 2e). Consistently, qRT-PCR analysis of selected mRNAs among those of UTR associated demonstrated a loss of expression (*RAB26*, *ASCC3*, *CAMK2B*, *NQO1*, *IGF2*, *HMOX1*, *ALDH2*, *PTBP2*, *RGS4*, *STC1*, *STY1*, *RTN1*) (Supplementary Figure S2d). Western analysis confirms down-regulation of corresponding protein expression of target mRNAs (*HMOX1*, *NQO1* and *CDH1*) on Star-PAP depletion (Figure 2g) consistent with the loss of Star-PAP association on the depletion. Further, 3′-RACE assay confirms the role of Star-PAP in the cleavage and polyadenylation of these mRNAs (*IGF2*, *COL5A1*, *BIK*, *KCNMA1*, *HMOX1*, *NQO1*) (Figure 2f). There was a loss of 3′-RACE product on Star-PAP depletion as reported earlier (Figure 2f). These results were further corroborated with cleavage assay where we observed increased accumulation of uncleaved pre-mRNAs (*KCNMA1*, *NQO1*, *COL5A1*, *WIF1*, *FEZ1*) while the expression levels were reduced on Star-PAP depletion (Figure 2h). Together, HITS-CLIP data confirm the global role of Star-PAP in the 3′-end processing of target mRNAs by the association at the 3′-UTR region. Interestingly, analysis of the polyadenylation site usage (PA-site choice) of these mRNAs revealed a higher distal PA-sites usage (~40%) consistent with earlier genome-wide Star-PAP APA analysis (Supplementary Figure S2f) [18]. We also observed proximal PA-site selection in around 30% of mRNAs whereas ~20% of mRNAs have single PA-sites (Supplementary Figure S2f). Functional analysis of these UTR-associated mRNAs shows a higher prevalence in cellular functions including cell cycle, apoptosis, myocyte hypertrophy, cell invasion, metastasis and metabolic pathways (Supplementary Figure S2g).

### 2.4. Star-PAP mRNA Binding Regulates Stability and Turnover Rate of Target mRNAs

**Figure 3:**
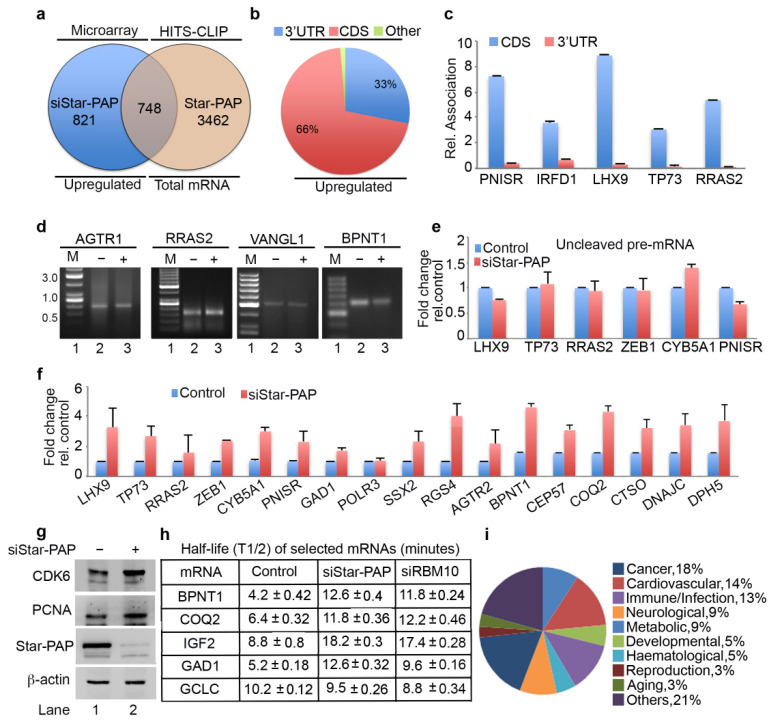
Star-PAP mRNA association regulates mRNA half-life in a novel Star-PAP-mediated mRNA metabolism pathway. (**a**) Venn diagram showing overlapped mRNA targets between up-regulated genes on siStar-PAP from earlier microarray analysis and mRNAs associated with Star-PAP detected in the Star-PAP HITS-CLIP experiment. (**b**) Distribution of Star-PAP associated nucleotide positions in the coding region and 3′-UTR region among up-regulated mRNAs on Star-PAP knockdown that is also common with that in Star-PAP HITS-CLIP experiment. (**c**) qRIP analysis showing the relative association of Star-PAP in the coding region and 3′-UTR region on select Star-PAP target mRNAs as indicated. Error bar represents SEM of three independent experiments. (**d**) 3′-RACE assay of various Star-PAP target mRNAs (*AGTR1*, *PNISR*, *VANGL1*, *BPNT1*) from total RNA isolated from HEK 293 cells from control or after Star-PAP knockdown as indicated. (**e**) Measurement of uncleaved pre-mRNA levels expressed relative to total mRNA after Star-PAP knockdown or control cells as indicated. (**f**) qRT-PCR analysis of select Star-PAP target mRNAs primarily associated at the coding region from HITS-CLIP experiment with total RNA isolated from HEK 293 cells with control and Star-PAP knockdown. (**g**) Western blot analysis of Star-PAP, target proteins, and control actin from control and Star-PAP knockdown HEK 293 cells. (**h**) Half-life (T1/2) measurement of various Star-PAP target mRNAs after transcription inhibition with actinomycin D under conditions as indicated. T1/2 is expressed in hours. Data are mean ± SEM of *n* = 3 independent experiments. (**i**) A schematic pie chart showing functional pathway analysis of Star-PAP target mRNAs obtained from HITS-CLIP experiment that were up-regulated on siStar-PAP microarray.

Among the up-regulated genes on Star-PAP depletion in our microarray, only around 50% of the genes were detected in Star-PAP HITS-CLIP (Figure 3b). These mRNAs represent the set of mRNAs whose expression is negatively regulated by Star-PAP binding. The other set of up-regulated mRNAs in the microarray (not detected in our Star-PAP HITS-CLIP) is likely controlled indirectly. Interestingly, among the Star-PAP targets detected in HITS-CLIP that are up-regulated on Star-PAP depletion (708 mRNAs), Star-PAP was mapped primarily at the CDS exonic regions (~70%), while a minority (<20%) was mapped in the 3′-UTR region (Figure 3b) suggesting a novel function of Star-PAP independent of 3′-end processing. Consistently, there was an overall higher coverage of the CDS region compared to the 3′-UTR region among these mRNAs (Supplementary Figure S3a). Further, qRIP analysis on select mRNAs (*PNISR*, *IRFD1*, *LHX9*, *TP73*, *RRAS2*) demonstrated primary association of Star-PAP at the CDS region over the 3′-UTR region on these mRNAs (Figure 3c). Consistently, 3′-RACE and cleavage assay show no effect of Star-PAP knockdown on the cleavage and polyadenylation of this set of mRNAs (Figure 3d,e).

To understand the mechanism of how Star-PAP binding negatively regulates the expression of these of mRNAs, we carried out the qRT-PCR analysis of select mRNAs (*LHX9*, *TP73*, *RRAS2*, *ZEB1*, *CYB5A1*, *PNISR*, *GAD1*, *POLR3*, *SSX2*, *RGS4*, *AGTR2*, *BPNT1*, *CEP57*, *COQ2*, *CTSO*, *DNAJC* and *DPH5*) (Figure 3f). We observed increased mRNA levels on Star-PAP depletion consistent with our microarray analysis. Western blot also showed increased protein levels on Star-PAP depletion (Figure 3g) suggesting Star-PAP’s role as a negative regulator of mRNA stability (Figure 3f,g). To further understand the mechanism, we measured mRNA stability and turnover by measuring half-life (*BPNT1*, *COQ2*, *IGF2*, *GAD1* and control non-target *GCLC*) after inactivating transcription with actinomycin D in the presence and absence of Star-PAP knockdown (Figure 3h). Strikingly, there was an increase in the half-life (2 to 3-fold) of mRNAs with no effect on the non-target *GCLC* mRNA (Figure 3h). These results demonstrate that Star-PAP binding on the CDS region de-stabilises mRNA and as a result a depletion of Star-PAP results in both increased mRNA and protein expressions. A list of Star-PAP-associated mRNAs down-regulated on siStar-PAP is shown in Supplementary Table S2. Functional pathway analysis of these mRNAs showed enrichment of genes involved in diseases including cardiovascular, metabolic, infection, and cancer (Figure 3i). A list of Star-PAP associated mRNAs up-regulated on Star-PAP knockdown is shown in Supplementary Table S2.

### 2.5. Transcriptome-Wide Star-PAP Binding Analysis after RBM10 Depletion Indicates Global Role of RBM10 in Star-PAP Target mRNA Association

**Figure 4:**
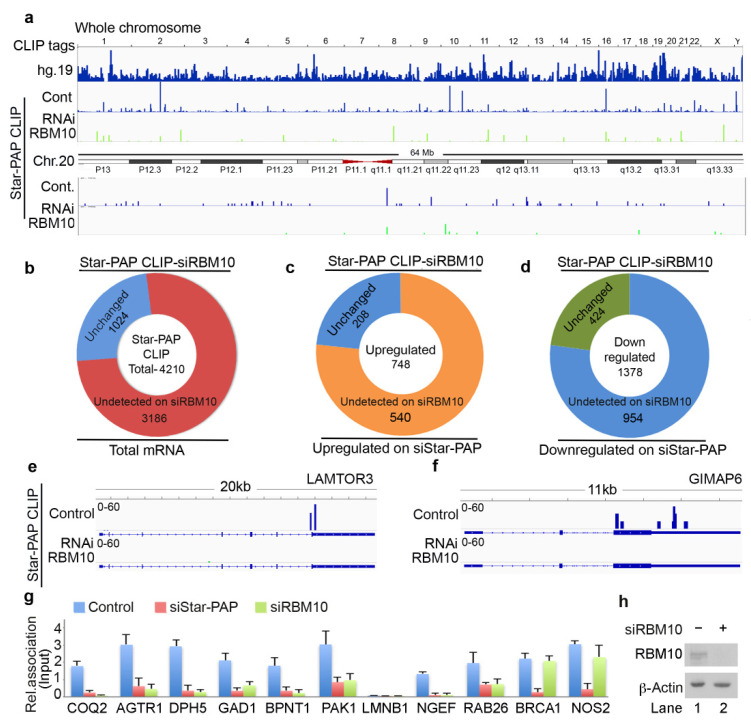
Star-PAP global mRNA association requires co-regulator RBM10. (**a**) HITS-CLIP signals of Star-PAP distribution across human genome showing loss of whole chromosomal association of Star-PAP on RBM10 knockdown in HEK 293 cells. Reference genome hg19 is indicated on top. Star-PAP HITS-CLIP tag distribution in one of the chromosome (chromosome 20) and the loss of the mapped tags on siRBM10 is shown below. The CLIP tags were counted after peak calling. (**b**) Doughnut plots showing the total number of Star-PAP target mRNAs that were not detected after RBM10 depletion. (**c**,**d**) Doughnut plots showing up-regulated and down-regulated mRNAs on Star-PAP depletion that were not detected in the Star-PAP HITS-CLIP after RBM10 depletion. (**e**,**f**) Star-PAP HITS-CLIP read cluster association on select target mRNAs in the presence and absence of RBM10 knockdown as indicated. (**g**) qRT-PCR analysis of Star-PAP target mRNAs that are detected in our HITS-CLIP in the presence and absence of Star-PAP and RBM10 knockdowns. (**h**) Western blot analysis of RBM10 in control and RBM10 knockdown in HEK 293 cells.

RBM10 is a Star-PAP-associated protein that is required for the regulation of mRNAs involved in cardiac hypertrophy [24]. Therefore, we investigated the genome-wide role of RBM10 in the Star-PAP recognition of target mRNAs. We carried out a similar HITS-CLIP experiment of Star-PAP after siRNA-mediated depletion of RBM10 in HEK 293 cells (Figure 4a). Strikingly, RBM10 depletion resulted in a loss of >60% of mapped read clusters associated with different chromosomes (Figure 4a, Supplementary Figure S3b). Relative reductions of Star-PAP association on six select chromosomes on RBM10 depletion are shown in Supplementary Figure S3b. Among the 4200 protein-coding genes bound by Star-PAP detected in our HITS-CLIP, ~70% of the mRNAs were not detected after RBM10 knockdown (Figure 4b). Interestingly, among the down-regulated genes on Star-PAP knockdown (UTR regulated), the majority (around 950) of mRNAs were not detected after RBM10 depletion (Figure 4d) indicating that RBM10 is required for Star-PAP association on target PA-sites. Moreover, among the >700 up-regulated genes on siStar-PAP that are detected on HITS-CLIP (negative regulation by Star-PAP), the majority (~540 mRNAs) were not detected after the RBM10 depletion (Figure 4c). This indicates the role of RBM10 on an overall Star-PAP target mRNA association. A list of mRNAs in Star-PAP HITS-CLIP lost on RBM10 depletion is tabulated in Supplementary Table S3. Four select mRNAs where there was a loss of Star-PAP association on RBM10 depletion are shown in Figure 4e,f and Supplementary Figure S3c,d. Together, these results indicate that Star-PAP target mRNA association requires RBM10 in the cell.

### 2.6. RBM10-RNA Association Regulates Star-PAP-Mediated mRNA Metabolism

**Figure 5:**
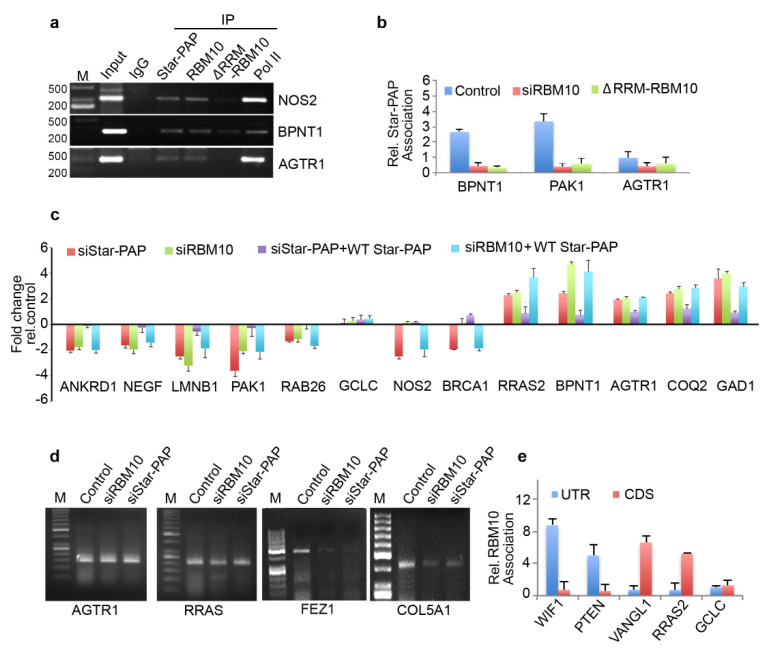
RBM10 regulates Star-PAP mediated mRNA metabolism. (**a**) RIP analysis of Star-PAP, RBM10, and RBM10 RNA binding motif deletion along with control RNA pol II on various Star-PAP targets in control and siRNA treated HEK 293 cells as indicated. Input, 10% of the IP lysate. M, marker lane. (**b**) qRIP analysis of Star-PAP association with various altered mRNAs as indicated in the presence of control or RBM10 siRNA and RRM deleted RBM10 in HEK 293 cells. (**c**) qRT-PCR analysis of various Star-PAP target mRNAs in the presence of Star-PAP or RBM10 depletion with the rescue with wild type Star-PAP ectopic expression in HEK 293 cells. (**d**) 3′-RACE assay of various Star-PAP target mRNAs from total mRNAs isolated after siStar-PAP or siRBM10 in HEK 293 cells. (**e**) qRIP analysis showing the relative association of RBM10 in the coding region and 3′-UTR region on select Star-PAP target mRNAs as indicated. Error bar represents SEM of three independent experiments.

The genome-wide loss of Star-PAP association on RBM10 depletion was further tested using qRIP experiment using 10 select mRNAs (*COQ2*, *AGTR1*, *DPH5*, *GAD1*, *BPNT1*, *PAK1*, *LMNB1*, *NGEF*, *RAB26*, *BRCA1*, *NOS2*) (Figure 4g). We selected both sets of mRNAs that were up-regulated or down-regulated on Star-PAP depletion. We observed a clear loss of Star-PAP association in all mRNAs investigated upon RBM10 depletion (Figure 4g). There was no effect of RBM10 knockdown on RBM10 independent Star-PAP target mRNAs. Western analysis for siRNA depletion of RBM10 is shown in Figure 4h. This confirms the requirement of RBM10 for Star-PAP target mRNA binding. Similarly, in a RIP analysis, we observed the association of both RBM10 and Star-PAP on Star-PAP target mRNAs (*AGTR1*, *BPNT1*, *NOS2*) and that Star-PAP association was a loss on RBM10 depletion (Figure 5a) indicating that RBM10 RNA binding is required for Star-PAP association with the target mRNA. To confirm this, we tested Star-PAP association with target RNAs with RBM10 RNA binding motif deletion (that compromised RBM10 RNA binding) (Figure 5b). For this purpose, we ectopically expressed wild type and RNA binding motif deleted RBM10 that has silent mutations on the targeting siRNA sites. We observed a significant loss of Star-PAP association with target RNAs (*BPNT1*, *PAK1*, *AGTR1*) on Star-PAP knockdown as well as RRM motif deletion RBM10 revealing that RBM10 binding is required for the Star-PAP association with the target RNA (Figure 5b). RBM10 is a U-rich or G-rich sequence binding protein and therefore, we tested the nucleotide sequence on mRNAs with Star-PAP mapped regions that were lost on RBM10 depletion. We observed a higher U-content and G-content of Star-PAP reads on mRNAs where Star-PAP was not detected on RBM10 depletion (Supplementary Figure S4a). Moreover, analysis of motif at these reads by CentriMo software indicates the potential association of 6U binding motif with a frequency of 48%, 7U with 33% and 8U with 18%, respectively, but a marginal possibility of G-motifs with 10% for 6G, 5% for 7G and <3% for 8G, respectively (Supplementary Figure S4b). Together, these results indicate that the RBM10 RNA association regulates Star-PAP target mRNA binding.

Therefore, we tested mRNA metabolism from both sets of mRNAs (down-regulated and up-regulated on Star-PAP depletion from our microarray analysis). First, RBM10 knockdown resulted in a differential expression of Star-PAP target mRNAs—a loss of expression of a set of mRNAs (*ANKRD1*, *NEGF*, *LMNB1*, *PAK1*, *RAB26*, *NOS2*) (UTR-associated) whereas an increased expression for another set of RNAs (*RRAS2*, *BPNT1*, *AGTR1*, *COQ2*, *GAD1*) (CDS-associated) (Figure 5c). The altered expression on RBM10 depletion was not rescued by Star-PAP ectopic expression (Figure 5c). This indicates that RBM10 is involved in the Star-PAP-mediated mRNA metabolism. Since the UTR-associated mRNAs were regulated through 3′-end processing and the CDS-associated group through RNA turnover and stability, we tested both 3′-UTR RNA processing (by cleavage assay and 3′-RACE assay) and RNA half-life measurement. In a 3′-RACE assay, there was compromised mRNA maturation on RBM10 depletion on *FEZ1* and *COL5A1*, whereas, there was no effect of RBM10 depletion on *AGTR1* or RRAS (up-regulated on the knockdown) (Figure 5d). Similarly, in the cleavage assay, RBM10 knockdown affected specifically mRNAs that were compromised on Star-PAP knockdown with no effect on the RNAs that were up-regulated on Star-PAP depletion (Supplementary Figure S4c,d). Concomitantly, measurement of half-life after RBM10 knockdown indicated increased half-life of target mRNAs similar to Star-PAP depletion (Figure 3h). Consistently, there was an overall higher RBM10 association at the 3′-UTR of down-regulated mRNAs whereas CDS association was prominent for the up-regulated mRNAs (Figure 5e). Together, these results indicate that RBM10 is required for Star-PAP mediated mRNA metabolism in both 3′-end processing and RNA destabilisation. Interestingly, among the RBM10-independent mRNAs (mRNAs where Star-PAP association was not affected by RBM10 depletion), Star-PAP was largely associated with the CDS region (~66% of the mRNAs) compared to the UTR region (~24% of the mRNAs) (Supplementary Figure S4e). Similarly, these mRNAs also exhibited higher proximal PA-site (40%) usage than the distal PA-site (25%) usage as opposed to the RBM10 dependent mRNAs (Supplementary Figure S4f) suggesting a role of RBM10 in Star-PAP mediated APA. Functionally, these mRNAs show enrichment of signalling pathways including RTK-MAPK, PI3K-Akt, JAK-STAT, mTOR and TGF-β in the cell (Supplementary Figure S4g).

## 3. Discussion

Star-PAP is a variant PAP that plays a critical role in the 3′-end processing of select mRNAs [20,21,25]. Star-PAP follows a distinct processing mechanism that is dispensable of important canonical components including CstF-64. Star-PAP instead requires additional associated factors including kinases and RNA binding proteins [1,34]. Star-PAP binds to target mRNA UTR and helps recruit the cleavage and polyadenylation factors [20,34]. However, the role of Star-PAP-associated factors in the Star-PAP UTR/PA-site selection or in the processing reaction is unclear. From mass spectrometry analysis, we established RBM10 as a unique Star-PAP coregulator required for specific mRNA regulation involved in myocyte hypertrophy [24]. In this study, we showed that RBM10 regulates global Star-PAP association on target mRNAs. This is consistent with the ubiquitous expression patterns of both the proteins where RBM10 will be required for Star-PAP mediated 3′-end processing [21,24]. Nevertheless, our study strongly indicates the role of RBM10 in determining Star-PAP specificity. There are two aspects of Star-PAP specificity: first, the selection of a PA-site, and second, the exclusion of canonical PAP from the target PA-site to have an exclusive/specific control of targets by Star-PAP [1]. RBM10 can have roles in both these aspects of specificity. In the first aspect, the RBM10-RNA association would recruit Star-PAP in a sequence-specific manner to help assemble a stable Star-PAP cleavage complex. In line with this, a loss of RBM10 would compromise Star-PAP binding on the RNA as observed in our study. Second, RBM10 binding at the vicinity of the Star-PAP binding region could exclude canonical PAPα or other components of canonical machinery that are absent from the Star-PAP processing complex. This supports our earlier hypothesis that Star-PAP requires a co-regulator for the function and specificity of its cellular activities [1]. Such specificity driven by associated factors will have important ramifications in the regulation of Star-PAP mediated alternative polyadenylation [18,23]. Yet, the role of RBM10 in APA is yet to be defined.

We reported a GC-rich sequence with an -AUA- motif for Star-PAP recognition, and a suboptimal downstream region with a U-depleted sequence on Star-PAP targets [20]. We confirmed from our HITS-CLIP experiment that Star-PAP-associated regions have a biased GC over AU composition in addition to a motif containing AUA on global Star-PAP targets. While sequence specificity for Star-PAP is critical, earlier reports indicate the signalling regulations are critical for the Star-PAP specificity [23,25,50,51]. Such signalling influence on specificity may operate through associated proteins such as RBM10. At least three agonists-oxidative stress, hypertrophic signal, and the toxin dioxin are known to regulate Star-PAP target mRNA selection [21,23,24,50]. It is still unclear how these signals drive the Star-PAP functions. Our finding of the RBM10 requirement for Star-PAP association shows the potential involvement of RBM10 in transducing the signal-mediated specificity of Star-PAP targets. This is consistent with RBM10′s role in the regulation of Star-PAP target anti-hypertrophy regulators in the heart [24]. Similarly, kinases CKIα/ε and PKCδ are also shown to modulate Star-PAP mRNA selection [25,50,51]. This could occur through either direct Star-PAP phosphorylation or indirectly via RBM10 phosphorylation that affects the sequence-specific binding of Star-PAP on distinct mRNAs. One of the phosphorylations at the ZF region on Star-PAP (Serine 6) was shown to regulate the specificity of Star-PAP regulation of some mRNAs involved in stress response and cell invasion [49,51]. Yet, the overall sequence-specific changes for Star-PAP induced by signalling conditions or by different phosphorylation statuses are yet to be defined.

**Figure 6:**
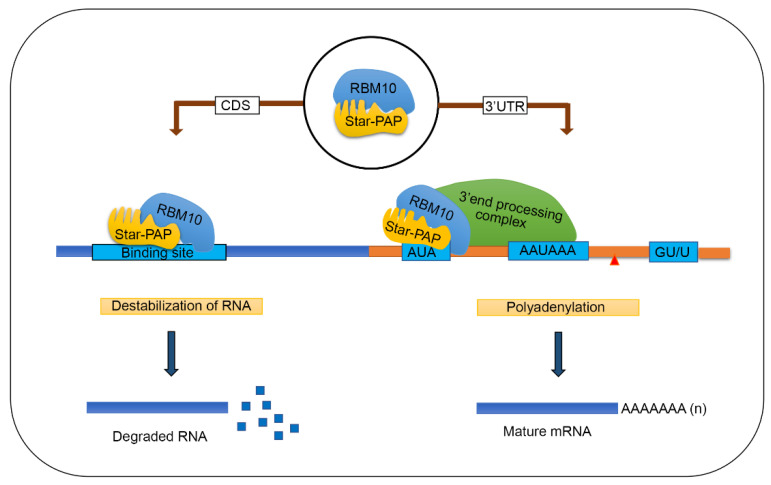
A model showing how RBM10 regulates Star-PAP-RNA association and mRNA metabolism (mRNA 3′-end processing and mRNA de-stabilisation).

Star-PAP has an established role in the 3′-end RNA processing that controls the expression of a large number of mRNAs that regulate various cellular functions [23,25,26,27,49,51]. In addition to its adenylation function, Star-PAP has a confirmed uridylation activity [28,52]. The substrate preference of Star-PAP (U vs. A) in the cell is likely driven by associated factors or co-regulators such as RBM10 [20,21,24]. Additionally, Star-PAP has also been shown to regulate the stability and processing of miRNAs [26,30,31,53]. The depletion of Star-PAP resulted in a decrease in the levels of a large number of miRNAs, yet how Star-PAP regulates miRNA expression is unclear [30]. Star-PAP can be immunoprecipitated with specific miRNAs and also along with the RISC complex proteins indicating a potential post-transcriptional role on miRNA biogenesis [26,31]. Consistent with this, we also detected a number of miRNA associations with Star-PAP in our HIT-CLIP experiment. Together, these findings show a diverse role of Star-PAP in different RNA processing events. In this study, we show a new function of Star-PAP in the mRNA metabolism that regulates mRNA stability and/or turnover. A model of how RBM10 regulates Star-PAP-RNA association and mRNA metabolism is shown in Figure 6. Here, Star-PAP acts as a negative regulator and its binding destabilises target mRNAs. This function is independent of Star-PAP polyadenylation of target mRNAs, uridylation of U6 snRNA, and miRNA regulations [21,30,52]. Nevertheless, this affects more than 1000 mRNA targets involved in multiple cellular functions and signaling pathways. RNA binding proteins are known to regulate the stability of the bound RNA (e.g., ARE binding proteins HNRNPD, ZFP36, TTP, KSRP or BRF5) that can promote mRNA turnover via recruiting decapping enzyme at the 5′-end or recruiting deadenylating enzyme at the 3′-end [54,55,56,57,58]. Alternatively, Star-PAP could promote mRNA silencing by binding near AGO2 sites and contributing to its loading with miRNAs as in the case of AUF1 protein [59,60,61]. Star-PAP is known to interact with AGO2 and also pull down miRNA [26,31]. Therefore, Star-PAP binding could also promote miRNA-mediated silencing on the Star-PAP-associated target mRNAs by recruiting targeting miRNAs.

## 4. Materials and Methods

### 4.1. Cell Culture, Transfections and Treatment

HEK 293 cells were obtained from American Type Cell Culture Collection. HEK 293 cells were maintained in Dulbecco’s Modified Eagle’s Medium (Himedia, Mumbai, India) with 10% Foetal Bovine Serum (Gibco Biosciences, Dublin, Ireland) and 50 U/mL Penicillin Streptomycin (Gibco) at 37 °C in 5% CO_2_. Transient knockdown experiments were carried out using custom-made siRNAs (Eurogenetec, Seraing, Belgium) by calcium phosphate method as described earlier [23]. Transient overexpression of Star PAP and RBM10 were performed using pCMV Tag2A constructs expressing FLAG-epitope tagged Star-PAP and RBM10 that has silent mutations rendering the siRNA used for the knockdown ineffective as described earlier [24,50]. Whenever required, cells were treated with actinomycin D (5 µg/mL in DMSO) and DMSO treatment was used as solvent control.

### 4.2. RNA Isolation

Cultured HEK 293 cells from 10 cm dishes (1 mL/1 × 10^6^ cells) were harvested in 2 mL epi tubes. Harvested cells were then washed with PBS and total RNA was isolated using RNase easy mini Kit (Qiagen, Germantown, MD, USA) as per the instruction of the manufacturer.

### 4.3. Quantitative Real-Time PCR (qRT-PCR)

qRT-PCR was carried out in a CFX96 multi-colour system (Bio-Rad, Hercules, CA, USA) using iTaq SYBR Green Supermix (Bio-Rad, Hercules, CA, USA) as described previously [23]. Then, 2 µg of total RNA was reverse transcribed using MMLV reverse transcriptase (Invitrogen, Waltham, MA, USA) with oligodT primer. Real-time primers were designed using Primer3 software and the difference in the melting temperature of corresponding forward and reverse primers were less than 1. Melt-curve analysis was used to check for single-product amplification and primer efficiency was near 100% in all experiments. Quantifications were expressed in arbitrary units, and target mRNA abundance was normalised to the expression of *GAPDH* with the Pfaffl method [62]. All qRT-PCR results are representative of at least three independent experiments (*n* > 3).

### 4.4. Cleavage Assay

To determine the cleavage efficiency of mRNA, the accumulation of non-cleaved mRNA levels was measured by quantitative real-time PCR (qRT-PCR). Total RNA was reverse transcribed using random hexamers and qRT-PCR was carried out using a pair of primers across the cleavage site to amplify non-cleaved mRNAs as described earlier [21]. Non-cleaved messages were expressed as fold-change over the total spliced mRNA.

### 4.5. HITS-CLIP Sequencing and Analysis

HITS-CLIP experiments were carried out as described earlier [63,64]. Briefly, HEK 293 cells grown on 15 cm plates were UV irradiated (400 mJ/cm^2^) three times for 15 min each before harvesting. Cells were then harvested and lysed in 1X PXL buffer (1× PBS, 0.1% SDS, 0.5% deoxycholate, 0.5% NP-40, and protease inhibitor Cocktail) by sonication. The lysate was then treated with DNase I followed by partial RNAse digestion. Debris was then separated by high ultracentrifugation at 30,000 rpm for 40 min at 4 °C. Next, immunoprecipitation was carried out from the supernatant using anti-Star-PAP antibody [50] conjugated with pre-incubated Dynabeads Protein A (Invitrogen, Waltham, MA, USA) in the presence of bridging anti-rabbit IgG antibody, overnight at 4 °C. IP samples were washed twice with 1X PXL, followed by 5× PXL high salt wash buffer (5× PBS, 0.1% SDS, 0.5% deoxycholate, 0.5% NP40) and three times with 1× PNK buffer (50 mMTris-HCl, pH 7.4, 10 mM MgCl_2_, 0.5% NP40). Washed IP samples were further treated with PNK (80 µL PNK reaction) with 10× PNK buffer, 4 µL of PNK enzyme and 1 µL of ATP) in a thermomixer at 37 °C for 20 min. It was further washed with 1× PXL and 5× PXL and twice with 1× PNK buffer. The efficiency and specificity of IP were confirmed by Western blot analysis and denaturing acrylamide gel. Protein–RNA complexes were subjected to Proteinase K (Sigma-Aldrich, St. Louis, MO, USA) digestion at 37 °C, 20 min in 1× PK/urea buffer and the released RNA fragments were extracted by acid Phenol:Chloroform:Isoamyl alcohol. It was followed by overnight precipitation using 3 M sodium acetate and 0.75 µL glycogen and 1:1 of ethanol: isopropanol. CLIP RNA fragments were finally resuspended in 10 mM Tris-HCl pH 7.5. Library preparation and sequencing were carried out at the commercially available facility at Genotypic Technologies (http://www.genotypic.co.in, accessed on 7 September 2017). The library was prepared using NEBNext Ultra Directional RNA Library Prep kit as per the manufacturer’s instruction and sequenced on an Illumina platform.

The raw data generated was checked for quality using FastQC (https://www.bioinformatics.babraham.ac.uk/projects/trim_galore/, accessed on 20 May 2019). Low-quality sequences, artifacts sequences, contaminated sequences and low-quality reads were filtered using Clip tool kit (fastq_filter.pl, 20 May 2019) (mean score 20 from 0–24) [65]. Cutadapt was used to trim low-quality sequences from the ends before the adapters and to remove universal adapter sequences (DOI:10.14806/ej.17.1.200). Filtered and trimmed sequences were then subjected to duplicate removal using Cliptool Kit (fastq2collapse.pl, 20 May 2019) [65]. Reads were then mapped to the human reference genome (hg19) using Burrows–Wheeler Aligner (BWA) tool [66]. The MAPQ score and the minimal mapping size (in parseAlignment.pl program of Cliptool kit) were set to 1 and 18 nt, respectively, so that a single read in alignment file should map to a single locus in genome [65]. The duplicate tags with the same start coordinates mapped on chromosomes at the 5′ end of RNA tags were collapsed together using Clip tool kit (tag2collapse.pl, 20 May 2019) [65]. Peak calling was then performed using Model-based Analysis of ChIP-Seq (MACS2) using IgG as control with the parameter set of high confidence enrichment ratio against a background with an mFold range of minimum 5 and maximum 50 against and fragment size of 500 and filtered the peaks using *p*-value 0.05 [67]. After processing with this parameter and IgG subtraction, we identified more than 420,000 read clusters. Genomic annotations were further obtained by extracting co-ordinates of the read clusters with reference human genome hg19 using Bedtools intersect [68]. Basic unix utilities (sort, uniq, awk and sed, etc.) were used for parsing and sorting based on genomic features. Next, the Integrative Genomics Viewer tool was used to visualize the genome-wide analysis, complete chromosomal visualisation peak and specific binding peaks in various regions of a gene [69]. Nucleotide compositions were then extracted using Galaxy (https://usegalaxy.org/, 10 October 2020) and plotted % nucleotide content as box plot. Motif detection software MEME-Chip was used to identify the binding motifs from the read sequences with E-value cut off of 0.05 [70].

### 4.6. RNA Immunoprecipitation (RIP)

RIP analysis was carried out as described earlier [21,71]. Briefly, HEK 293 cells were cross-linked with 1% formaldehyde for 10 min followed by the addition of 0.125 M glycine for 5 min to halt crosslinking. Washed cells were lysed by incubating for 10 min in 300 µL of cell lysis buffer (10 mM Tris-HCl pH 8.0, 10 mM NaCl, 0.2% NP-40, 1× EDTA free Proteinase Inhibitor, 1000 U RNaseI (Promega, Madison, WI, USA). Crude Nuclei pelleted (5 min, 2500 rpm at 4 °C) and 400 µL nuclei lysis buffer (50 mM Tris-HCl pH 8.0, 10 mM EDTA, 1% SDS, 1× EDTA free Proteinase Inhibitor, 1000 U RNaseI (Promega, Madison, WI, USA)) added to the pellet and sonicated at 22% amplitude 20 s pulse for 5 min. The nuclear lysate was centrifuged at 15,000 rpm for 10 min, supernatant collected and treated with DNase1 for 20 min at 37 °C followed by the addition of EDTA to 20 mM to stop the digestion. Supernatant incubated overnight at 4 °C with respective antibodies for Star-PAP, RBM10, RNA Pol II, FLAG and Rabbit IgG. Further, the mixture was incubated with Protein G beads which were equilibrated with IP dilution buffer (20 mM Tris-HCl pH 8, 150 mM NaCl, 2 mM EDTA, 1% Triton X-100, 0.01% SDS, 1× EDTA free Proteinase Inhibitor, 1000 U RNaseI (Promega, Madison, WI, USA) for 2 h at 4 °C. Further, the complex was pelleted down at 5000 rpm for 5 min at 4 °C and washed with IP dilution buffer (3× 5000 rpm for 5 min at 4 °C). Immunoprecipitates were eluted with 300 µL elution buffer (1% SDS, 100 mM Sodium bicarbonate) and NaCl added to 200 mM followed by proteinase K for 2 h. Reverse crosslinking was then carried out by incubating the mixture at 67 °C for 4 h. RNA was isolated from the mixture using Trizol (Invitrogen, Waltham, MA, USA) reagent according to the manufacturer’s protocol. cDNA was then synthesised using Random Hexamers (Invitrogen, Waltham, MA, USA) by MMLV RT (Invitrogen, Waltham, MA, USA). PCR amplification was carried out and visualised on agarose gel.

For quantitative RIP, the immunoprecipitated RNA samples were diluted at 1:10. These samples along with input RNA were quantified using CFX multi colour system (Bio-Rad, Hercules, CA, USA) as described above. Values from each sample were corrected using reactions lacking reverse transcriptase. Quantifications were expressed in arbitrary units, and IgG immunoprecipitation product levels were used as controls for normalisation of the abundance of the target messages. The quantitative association was then expressed relative to the input RNA signal as described [72,73] using the method of Pfaffl [62]. The primers used for qRIP are listed in the Supplementary Text.

For determining UTR and CDS association on different mRNAs, we carried out qRIP analysis using specific primers designed at the 3′-UTR and CDS regions as described above.

### 4.7. Half-Life (T_1/2_) Measurement

HEK 293 cells were transfected with RNAi specific to Star-PAP and RBM10. Cells were then treated with actinomycin D (5 µg/mL in DMSO) for different time points (0, 2, 4, 6, 8, 12, 18, 24, 30, 36, 42 and 48 h) post-transfection. Cells were then harvested for total RNA was isolated from cells from each time point. cDNA was synthesised by oligodT primer followed by qRT-PCR as described above. mRNA half-life (T_½_) was measured as described earlier by following the decrease in % mRNA level over time with 0 time point taken as 100% of each mRNA expression [74].

### 4.8. 3′-RACE

3′-RACE assay was carried out as described previously [50]. Briefly, 2 µg of total RNA isolated from HEK 293 cell was used for cDNA synthesis using an engineered oligodT primer with a unique sequence at the 5′-end (Adapter primer) and MMLV-RT (Invitrogen, Waltham, MA, USA). This was followed by PCR amplification using a gene-specific forward primer and a universal adapter primer that is complementary to the unique sequence on the engineered oligodT primer (AUAP primer). The RACE products analysed on a 2% agarose gel and confirmed by sequencing.

### 4.9. Immunoblotting

Cell lysates were prepared in 1× SDS-PAGE loading buffer (0.06 M Tris, 25% Gylcerol, 2% SDS, 0.002% Bromophenol blue, 1% β-mercapto ethanol). Denaturation was carried out by heating the mixture at 95 °C for 20 min. Proteins were separated in SDS PAGE gel in 1× Tris Glycine Buffer (25 mM Tris pH 8.0, 190 mM Glycine, 0.1% SDS pH 8.3). Transfer of proteins to the PVDF was performed in a transfer buffer (25 mM Tris pH 8.0, 190 mM Glycine, 20% Methanol). PVDF membrane after transfer was blocked in 5% skimmed milk in 1× TBST (20 mM Tris pH 7.4, 150 mM NaCl, 0.1% Tween-20) for 45 min at room temperature. Primary antibodies were diluted in TBST as per the manufacturer’s instruction and incubated in a shaking platform overnight at 4 °C. The blots were washed in TBST 3 times for 10 min followed by incubation in HRP conjugated secondary antibody (Jackson Immuno Research Laboratory, West Grove, PA, USA). Further, imaging of the blots was carried out using chemiluminescent substrate (Bio-Rad, Hercules, CA, USA) in an iBright FL1500 platform (Invitrogen, Waltham, MA, USA).

### 4.10. Statistics

All data were obtained from at least three independent experiments and are represented as mean ± standard error mean, SEM. The statistical significance of the differences in the mean is calculated using ANOVA with statistical significance at a *p*-value of less than 0.05. All Western blots show representations of at least three independent blotting experiments.

### 4.11. Primers and Antibodies

A list of all the primers and antibodies employed in the study is shown in the Supplementary Data.

## Data Availability

The accession number for raw and processed HITS-CLIP data reported in this paper deposited at NCBI Gene Expression Omnibus (GEO) (https://www.ncbi.nlm.nih.gov/geo/) is GSE182643.

## Supplementary Materials

The following are available online at https://www.mdpi.com/article/10.3390/ijms22189980/s1.
