# Supplementary material for: Star-PAP RNA Binding Landscape Reveals Novel Role of Star-PAP in mRNA Metabolism That Requires RBM10-RNA Association
Source: Int J Mol Sci. 2021 Sep 15;22(18):9980. doi: 10.3390/ijms22189980 (PMC8469156; doi:10.3390/ijms22189980)
Supplement: Supplementary file 1 [file ijms-22-09980-s001.zip › ijms-1323973-supplementary proofed.pdf]

International Journal of *Molecular Sciences*

Supplementary data

Star-PAP RNA binding landscape reveals novel role of Star-PAP in mRNA metabolism that requires RBM10-RNA association

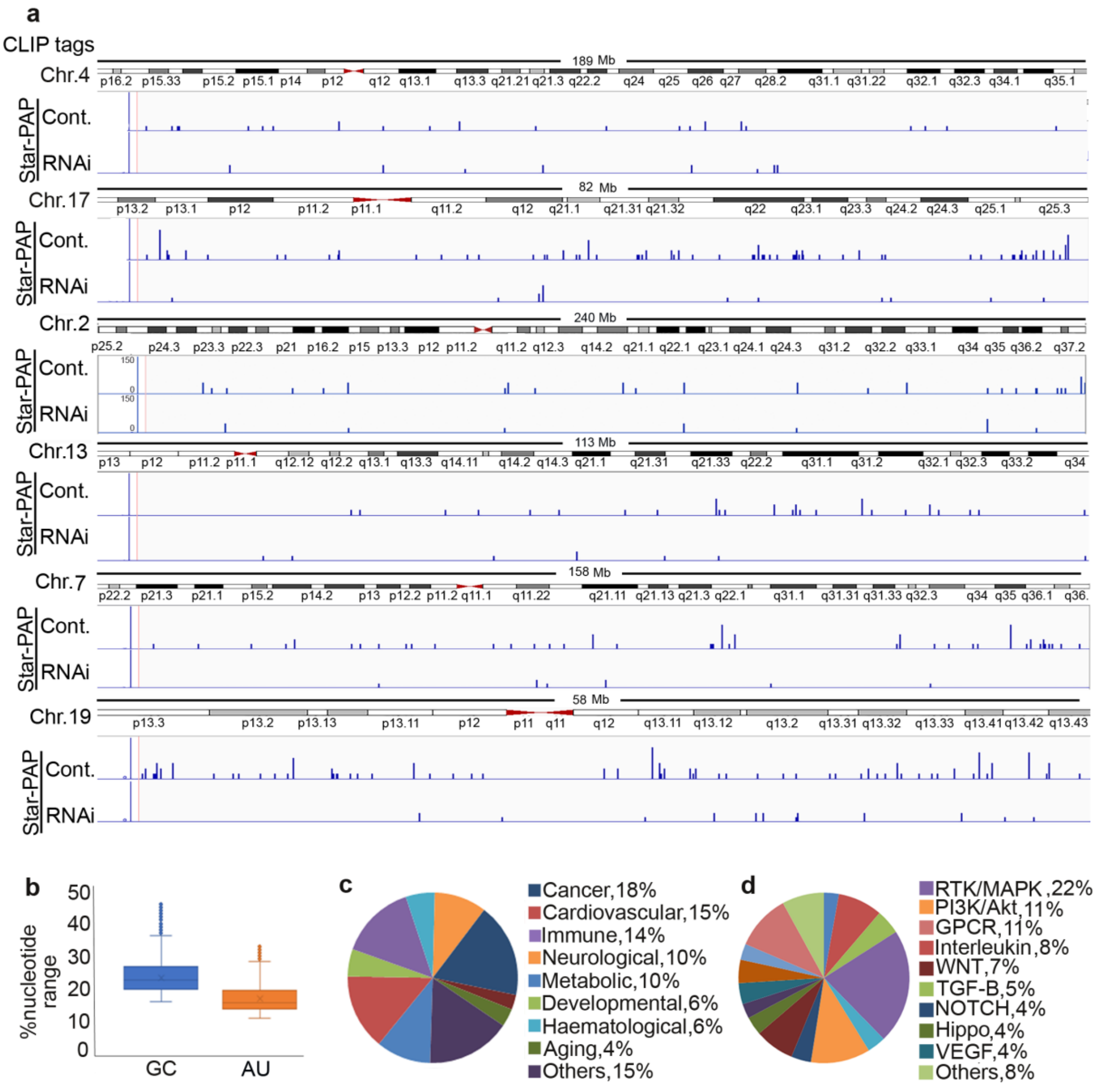

Figure S1. (**a**) Star-PAP HITS-CLIP reads cluster distribution and the loss of the mapped tags on siStar-PAP on different chromosomes as indicated. (**b**) Box plot showing %GC and AU nucleotide composition across Star-PAP mapped regions in the human genome. (**c**) A schematic pie chart showing functional pathway analysis of Star-PAP target mRNAs obtained from HITS-CLIP experiment. (A list of Star-PAP associated mRNAs is provided in Supplementary Table S1). (**d**) Signalling pathway analysis of Star-PAP target mRNAs that are detected in HITS-CLIP experiment. Percentage of genes in different signaling pathways is indicated.

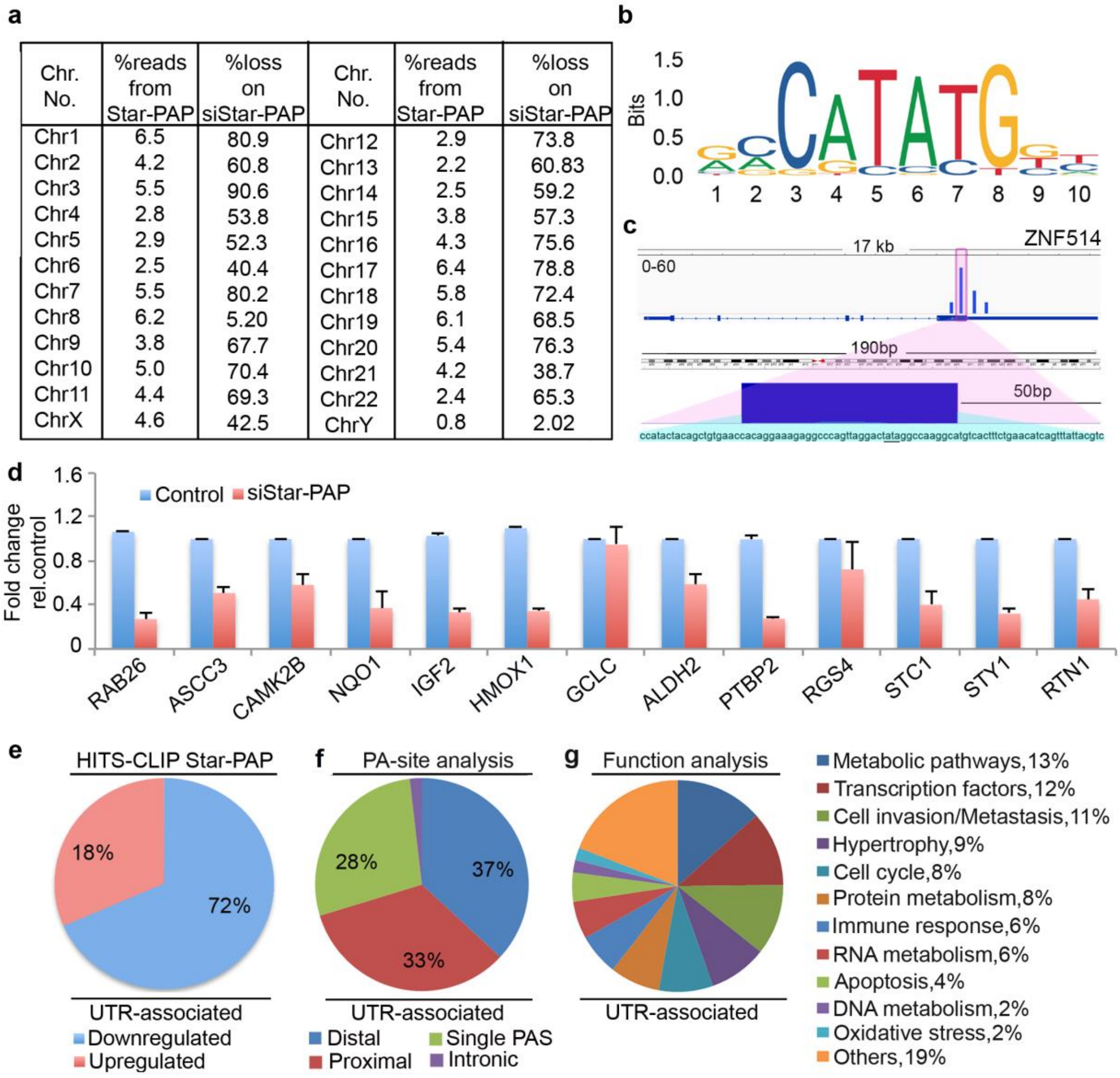

Chr. No.	%reads from Star-PAP	%loss on siStar-PAP	Chr. No.	%reads from Star-PAP	%loss on siStar-PAP
Chr1	6.5	80.9	Chr12	2.9	73.8
Chr2	4.2	60.8	Chr13	2.2	60.83
Chr3	5.5	90.6	Chr14	2.5	59.2
Chr4	2.8	53.8	Chr15	3.8	57.3
Chr5	2.9	52.3	Chr16	4.3	75.6
Chr6	2.5	40.4	Chr17	6.4	78.8
Chr7	5.5	80.2	Chr18	5.8	72.4
Chr8	6.2	5.20	Chr19	6.1	68.5
Chr9	3.8	67.7	Chr20	5.4	76.3
Chr10	5.0	70.4	Chr21	4.2	38.7
Chr11	4.4	69.3	Chr22	2.4	65.3
ChrX	4.6	42.5	ChrY	0.8	2.02

Figure S2. (**a**) % Star-PAP HITS-CLIP tags mapped in different chromosomes and % loss of the associated reads on Star-PAP knockdown on each chromosome. (**b**) In vivo Star-PAP binding motif obtained with MEME-Chip software using 20 nucleotide sequences surrounding the top 1000 Star-PAP associated cross-linked positions in the 3′-UTR and CDS regions. (**c**) Star-PAP HITS-CLIP association on select mRNA with distinct associations in 3′-UTR. An enlarged region at the peak area showing the associated nucleotide composition is shown below. (**d**) qRT-PCR analysis of Star-PAP target mRNAs from HEK 293 cells with control or Star-PAP specific siRNAs as indicated. (**e**) Comparisons of UTR associated Star-PAP target mRNAs with that of siRNA Star-PAP microarray analysis showing percentage of mRNAs down regulated or up regulated on siStar-PAP. (**f**) Analysis of PA-site choice on Star-PAP UTR associated mRNAs from Star-PAP HITS-CLIP. (**g**) Functional pathway analysis of same Star-PAP target mRNAs in f.

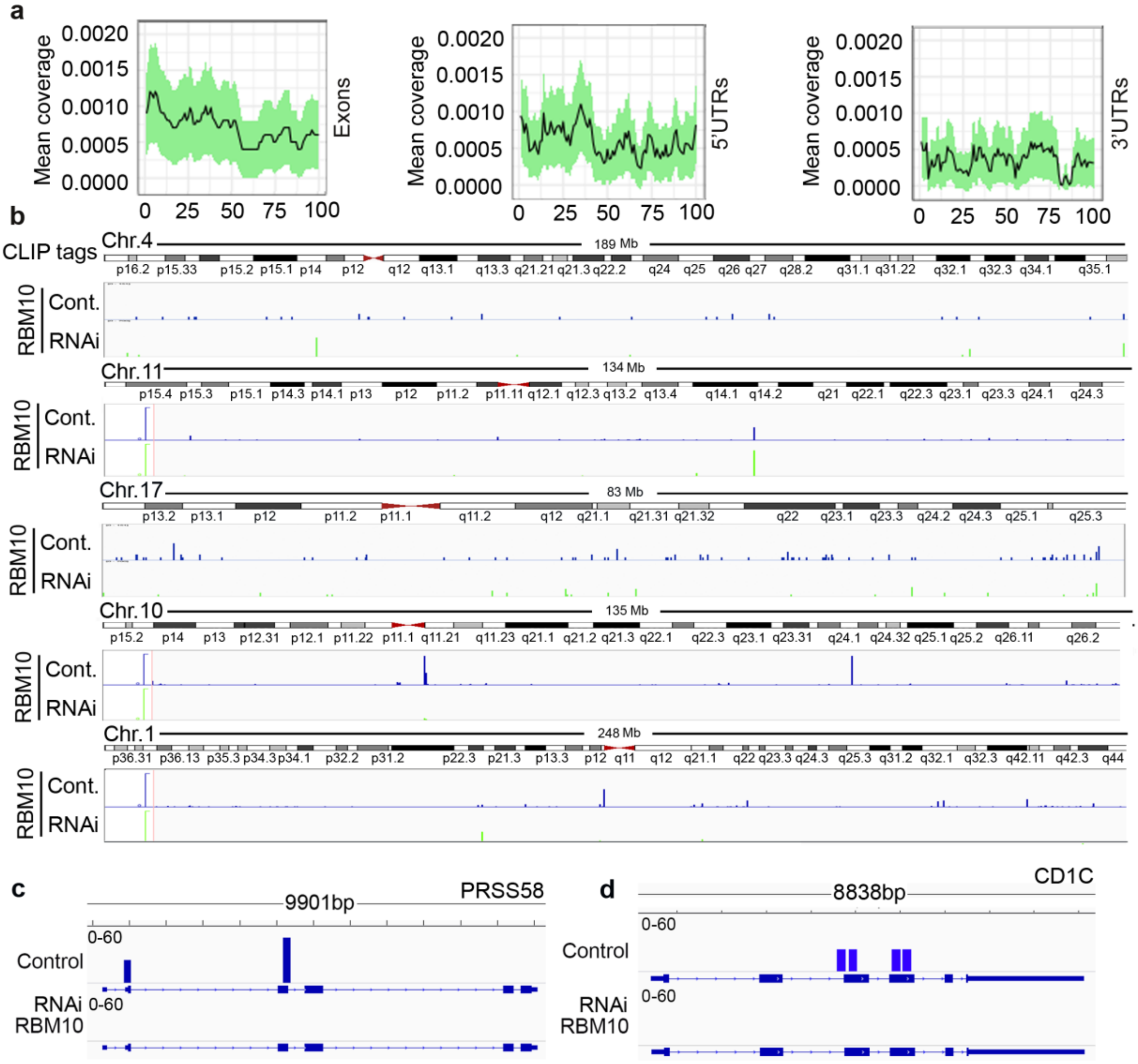

Figure S3. (**a**) Ribbon area plot showing mean coverage association of Star-PAP in 3′-UTR, 5′-UTR and exonic regions among the Star-PAP targets that are upregulated on siStar-PAP in microarray analysis. (**b**) Star-PAP HITS-CLIP tag distribution and the loss of the mapped read cluster on siRBM10 on different chromosomes as indicated. (**c**,**d**) Star-PAP HITS-CLIP association on select mRNAs in the presence and absence of RBM10 knockdown as indicated (distinct associations in the CDS or 5′-UTR regions are represented).

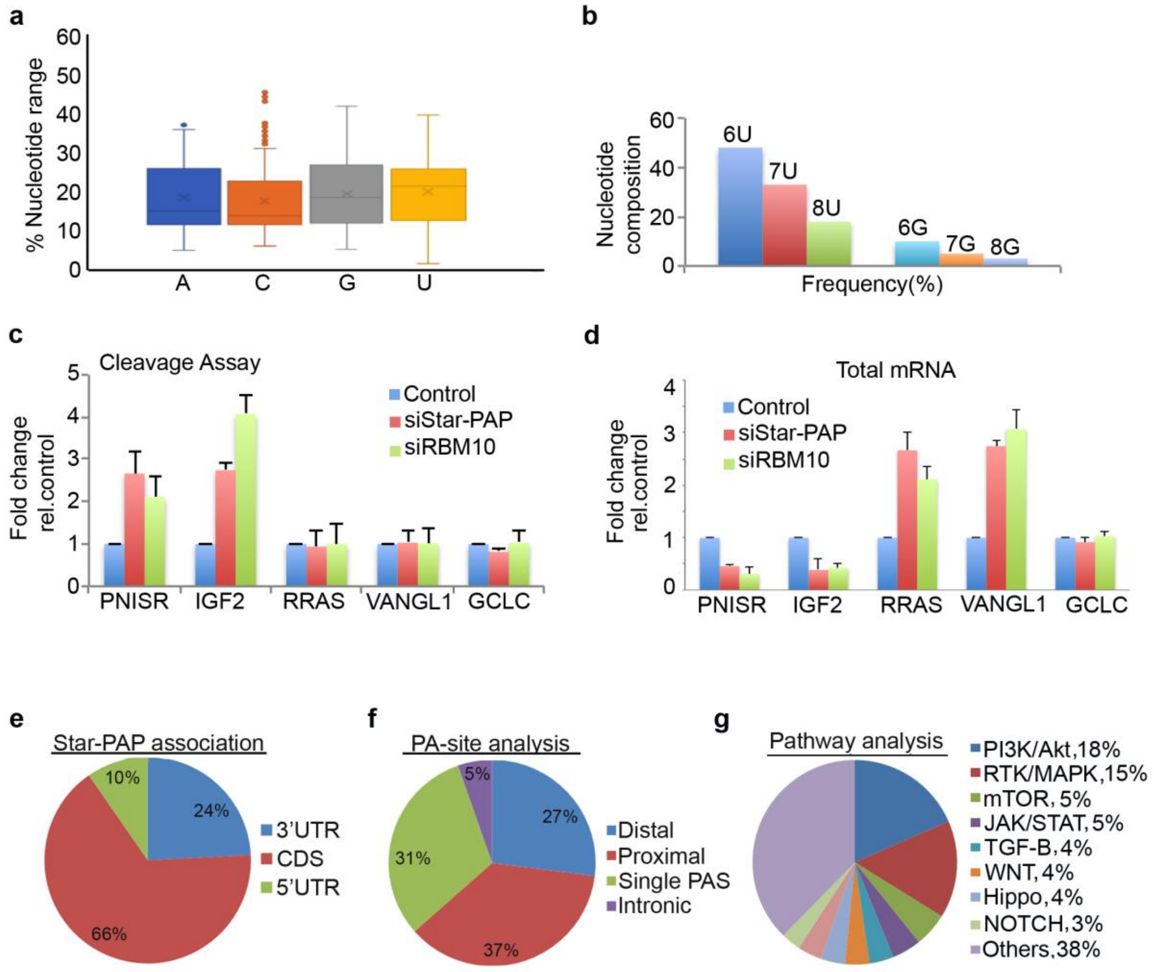

Scheme 4. (**a**) Box plot showing percentage nucleotide composition (G, C, A and U) across Star-PAP mapped regions at various genes that are lost on RBM10 knockdown. (**b**) Motif analysis using Centrimo (MEME) software on the nucleotide sequence at the Star-PAP mapped regions on mRNAs that were lost on RBM10 knockdown. Frequency of occurrence of 6U, 7U, 8U and 6G, 7G and 8G indicated. (**c**,**d**) Measurement of uncleaved pre-mRNA levels (**c**) expressed relative to total mRNA (**d**) after Star-PAP knockdown or RBM10 knockdown as indicated. (**e**) Distribution of Star-PAP associated nucleotide positions in coding region and 3′-UTR region of RBM10-independent Star-PAP associated mRNAs. (**f**) Analysis of PA-site choice on Star-PAP associated mRNAs that are independent of RBM10 depletion. (**g**) Signalling pathway analysis of same Star-PAP target mRNAs in (**f**).

List of antibodies

Mouse monoclonal anti-HMOX1 (A-3) antibody (Santacruz, TX, USA SC-136960), Mouse monoclonal β-actin (Santacruz SC-47778), Rabbit anti-RBM10 (Bethyl A301-006A), Rabbit anti-RNA polymerase II antibody (Bethyl, Montgomery, TX, USA A300-653A), Mouse monoclonal E-Cadherin (CDH1) antibody (Cell Signalling Technology, Danvers, MA, USA 24E10), Mouse monoclonal CDK6 antibody (Cell Signalling Technology, Danvers, MA, USA DCS83), Rabbit polyclonal anti-Star-PAP antibody (Mohan et. al. 2015).

List of primers employed in the study

qRT-PCR analysis primers

Gene	Forward (5′ to 3′ Direction)	Reverse (5′ to 3′ Direction)
KCNMA1	GAAGGTGATGACGCAATCTG	CTGCCATGTGTCTTCCTCAA
COL5A1	ATTCAAGCGTGGGAAACTG	TAGCAGTGGTAGGTGACGTTCT
NQO-1	GAACTTCAATCCCATCATTTCCAG	CAGCTTCTTTTGTTCAGCCACAAT
FEZ1	AACTTCCTCCCAGGCAGACT	CTGCTGCACCAGCTCCTC
RAB26	ATTCAAGGATGGTGCTTTCC	CACATCTGCAGCTTCACCTT
BPNT1	TGGGAGGCAAGTTAACCGAT	GCCCGGCCAAATGAAACTTT
CEP57	GGCATTAGTGGGAAGGATGG	CTGCTGCTGTTTCTTTCTTCA
COQ2	TGGTCTTAAGTCAACGGCTCT	GAGTCAGATGGGCTCCTACA
CTSO	GATTGTGCGGAATTCCTGGG	CTGCCCAACATGTCACACAA
DNAJC	AGCCAATACGAAGGGGAAGA	GGTGGAAGTGGGAGGAAAGA
DPH5	GCAGTTACCGAGGAGACACT	TTGGATGTATGCTGCCTCCT
GAD1	CTGAGCACACAAACGTCTGT	AATGTCAGACTGGGTAGCGG
POLR3F	GCAAAAGAAGGCACAGTTGG	TTCGAGCCACTCTGTCATGT
SSX21P	GTACTTCACCATCAACGGCG	GCCTTCTTGCCACCTTTCAG
RGS4	AAGTACGCTCAAAGCCGAAG	GTTGTGGGAAGAATTGTGTTCA
AGTR2	TGTGAACATCTCTGGCAACA	CCACAGCGAGGTTGAAGA
ASCC3	AGCCTTTGCCTTCCCAATAC	TTGGTAAAGGCTGAAGATCCA
CAMK2B	TCCTGAAGCCAAAAACCTCA	ATCATGGATGCTACCGTGGA
LHX9	CATCAACCACAACCCGGATG	AGTGGGATTGGTCAGGTCTG
TP73	TGAAGATCCCCGAGCAGTAC	GGTTGGGGATGGTGATGGT
RRAS2	AAGGACAACAGTTAGCACGG	TGGCCGTTGGTAGCTAAAAC
CYB5A	GAACTTTGAGGATGTCGGGC	TAGGCGATACATCAAGGCGA
PNISR	AGCAGCAAACAGAAAGGGTT	CTTCCCGACCTACTCCTTCC
WIF1	CAAGTTGGTTTCCCATGTCT	TTAAAGAAGATAGCATTTTGAGGTG
RTN1	CATCGTGTTTGGGAGTTTCC	AAGGGTGATCTCAAGCTCCA
SYT	TGACCAGGGACATCATCAAA	ACCGGTGATATCCATCTTCG
STC1	TTGCATGCCTGGAAAACTC	TCAGCAATCATCCTTTGGAA
PTBP2	GGCACCACAGTTAGCGAGAGTGCA	CTGGAGCAAAGCTTGAAACTGGTT
ALDH2	ACCTTCGTGCAGGAGGACAT	CGTGTTGATGTAGCCGAGGA
GCLC	AAGTTCTTGAAACTCTGCAAGAGAAGG	GCCTCAACTGTATTGAACTCGGAC
HO-1	CATGCAGCGCTATGTGAA	ATGTTGGGGAAGGTGAAGA
IGF2	TGGTGCTTCTCACCTTCTTG	GAAACAGCACTCCTCAACGA
BRCA1	TGTGCTTTTCAGCTTGACACAGG	CGTCTTTTGAGGTTGTATCCGCTG
NOS2	CTCAGCTCATCCGCTATGCT	TCAGGTGGGATTTCGAAGAG
AGTR1	TTGCCAGCTATAATCCATCG	CCTTCTTTAGGGCCTTCCAA
PAK1	AGTGTGGGCGATCCTAAGAA	TCCCTCATGACCAGGATCTC
LMNB1	GCTCTTGCTACTGCACTTGGT	GTAAGGCTCTGACAACGATTCTC
NGEF	TCACCTACGTCAGCAATCAG	TCTGGAAAGGCAGGATGA
ANKRD1	TGGATGTGCCTACGTTTCTG	GGCTCCAGCTTCCATTAACTT
ZEB1	GGCAGAGAATGAGGGAGAAG	CTTCAGACACTTGCTCACTACTC
GAPDH	GAAGGTCGGAGTCAACGGATTT	GAATTTGCCATGGGTGGAAT

RNA Immunoprecipitation andCDS/UTR binding analysis

Gene	Forward (5′ to 3′ Direction)	Reverse (5′ to 3′ Direction)
KCNMA1	GAAGGTGATGACGCAATCTG	CTGCCATGTGTCTTCCTCAA
COL5A1	ATTCAAGCGTGGGAAACTG	TAGCAGTGGTAGGTGACGTTCT
RRAS	GGTGTTCGCCATTAACGA	TGACTCCAGATCTGCCTTGT
VEGF	CTACCTCCACCATGCCAAGT	CACAGGATGGCTTGAAGATG
BIK	TCTTGATGGAGACCCTCCTG	GTCCTCCATAGGGTCCAGGT
AGTR1	TTGCCAGCTATAATCCATCG	CCTTCTTTAGGGCCTTCCAA
PTEN	AAGCTGGAAAGGGACGAACT	ACACATAGCGCCTCTGACTG

ZEB1	GGCAGAGAATGAGGGAGAAG	CTTCAGACACTTGCTCACTACTC
NQO-1	GAACTTCAATCCCATCATTTCCAG	CAGCTTCTTTTGTTCAGCCACAAT
FEZ1	AACTTCCTCCCAGGCAGACT	CTGCTGCACCAGCTCCTC
RAB26	ATTCAAGGATGGTGCTTTCC	CACATCTGCAGCTTCACCTT
BPNT1	TGGGAGGCAAGTTAACCGAT	GCCCGGCCAAATGAAACTTT
PNISR	AGCAGCAAACAGAAAGGGTT	CTTCCCGACCTACTCCTTCC
IFRD1		
GCLC	AAGTTCTTGAAACTCTGCAAGAGAAGG	GCCTCAACTGTATTGAACTCGGAC
PAK	AGTGTGGGCGATCCTAAGAA	TCCCTCATGACCAGGATCTC
NOS2	CTCAGCTCATCCGCTATGCT	TCAGGTGGGATTTCGAAGAG
GAPDH	GAAGGTCGGAGTCAACGGATTT	GAATTTGCCATGGGTGGAAT
KCNMA1 UTR	AAGTTACGCCCTCAGAACATTT	TTGGCCCATTCTATTCATCC
KCNMA1 CDS	TAGCAATATCCACGCGAACC	TGATGAGCGCATCCATCTT
NQO-1 UTR	TGCCTTCATCTTCACTGCAA	TTGTCAAGCCAGTCACCAAG
NQO-1 CDS	AGCCCAGATATTGTGGCTGA	TTGTCATACATGGCAGCGTAA
COL5A1 UTR	CTGTAATGCCCAGGAAAGGA	TTACGGTGGAAACACCCAAC
COL5A1 CDS	ATTCAAGCGTGGGAAACTG	TAGCAGTGGTAGGTGACGTTCT
WIF1 UTR	GAACTTTTTGCATTGGCTTGA	GTGCAAAGATCACCTCCACA
WIF1 CDS	CAAGTTGGTTTCCCATGTCT	TTAAAGAAGATAGCATTTTGAGGTG
GCLC UTR	ATGCCTGGTTTTCGTTTGCA	AGCTGTGGAACTCACACACACTCA
GCLC CDS	CATGATTGAAGGGACACCAG	TTGTGCAAAGAGCCTGATTT
FEZ1 UTR	CTGGCTACAGCCCTCAAAAA	AGAATGCTATACAGCCCGTCTC
FEZ1 CDS	AGCCCCAGTGTTTCTATGGTT	TTCCGAAAGCAGACATTGAG
RRAS2 UTR	ACAAAAGTGAACCATCTCAGACC	GAGTAGGAGTAGTAACACAGGGT
RRAS2 CDS	AAGGACAACAGTTAGCACGG	TGGCCGTTGGTAGCTAAAAC
VANGL1 UTR	CCCTTCCTCTTTCTCCCAGT	ACATTGTTCCCACTGCAAAAG
VANGL1 CDS	CAGAAGTACCTGCGCATCAC	TCATCACTGACAAGCCTCCA
LHX9 UTR	AGTGGTCTAGCAAAATTGTCCA	GCTGGAACACACTAGTACGG
LHX9 CDS	CATCAACCACAACCCGGATG	AGTGGGATTGGTCAGGTCTG
TP73 UTR	AGTTCAACCTAGCGCCCAT	AAGGAAAGCAATGCCCTGTG
TP73 CDS	TGAAGATCCCCGAGCAGTAC	GGTTGGGGATGGTGATGGT
PNISR UTR	AACATGCCATTCTCTTTCAGC	ACAAAATTCCAGCTGCCTTTT
PNISR CDS	AGCAGCAAACAGAAAGGGTT	CTTCCCGACCTACTCCTTCC
IFRD1 UTR	AGTGGTCTAGCAAAATTGTCCA	GCTGGAACACACTAGTACGG
IFRD1 CDS	GGAGGTTCTTGGATCAGGGA	GCTTTGAAGGCTGCAGAGTT
PTEN UTR	CCACAGGGTTTTGACACTTGT	ACACATCAGTCTGTCTCCACT
PTEN CDS	AAGCTGGAAAGGGACGAACT	ACACATAGCGCCTCTGACTG

Cleavage assay primers

Gene	Forward (5′ to 3′ Direction)	Reverse (5′ to 3′ Direction)
GCLC unclv	ATGCCTGGTTTTCGTTTGCA	AGCTGTGGAACTCACACACACTCA
GCLC total	CATGATTGAAGGGACACCAG	TTGTGCAAAGAGCCTGATTT
LHX9 unclv	AGTGGTCTAGCAAAATTGTCCA	GCTGGAACACACTAGTACGG
LHX9 total	CATCAACCACAACCCGGATG	AGTGGGATTGGTCAGGTCTG
TP73 unclv	AGTTCAACCTAGCGCCCAT	AAGGAAAGCAATGCCCTGTG

TP73 total	TGAAGATCCCCGAGCAGTAC	GGTTGGGGATGGTGATGGT
RRAS unclv	ATAAATGTCACTGCCAACGTCA	CTGGTCAAAAACTCCTGGACTC
RRAS total	AAGCAACCACCACAAGCTCT	AGGAAGTAAGGGTGGGTATGTG
ZEB1 unclv	GCCTGAACTGCTGTCATTCT	CCTCTCCAGAAACATTTAGCCA
ZEB1 total	GGCAGAGAATGAGGGAGAAG	CTTCAGACACTTGCTCACTACTC
NQO-1 UTR	TGCCTTCATCTTCACTGCAA	TTGTCAAGCCAGTCACCAAG
NQO-1 CDS	AGCCCAGATATTGTGGCTGA	TTGTCATACATGGCAGCGTAA
COL5A1 UTR	CTGTAATGCCCAGGAAAGGA	TTACGGTGGAAACACCCAAC
COL5A1 CDS	ATTCAAGCGTGGGAAACTG	TAGCAGTGGTAGGTGACGTTCT
WIF1 UTR	GAACTTTTTGCATTGGCTTGA	GTGCAAAGATCACCTCCACA
WIF1 CDS	CAAGTTGGTTTCCCATGTCT	TTAAAGAAGATAGCATTTTGAGG TG
CYB5A unclv	CTGTCCTTTCTGCGCTCAAA	CCAAACAGGCAAACACGGT
CYB5A total	GAACTTTGAGGATGTCGGGC	TAGGCGATACATCAAGGCGA
PNISR unclv	AACATGCCATTCTCTTTCAGC	ACAAAATTCCAGCTGCCTTTT
PNISR total	AGCAGCAAACAGAAAGGGTT	CTTCCCGACCTACTCCTTCC
IGF2 unclv	CATGCTTGGGTTTTGTCTTT	TTCCCCTTCCCTCTTCTATC
IGF2 total	TGACCTCATTTCCCGATACC	GGGGGAGAATTCGTCTGATT
VANGL1 cpFP	CCCTTCCTCTTTCTCCCAGT	ACATTGTTCCCACTGCAAAAG
VANGL1 total	CAGAAGTACCTGCGCATCAC	TCATCACTGACAAGCCTCCA
GAPDH unclv	CACACTGAATCTCCCCTCCT	TTGACACAAGCCCAGCTTC
GAPDH total	AGAACATCATCCCTGCCTCT	GCTTCACCACCTTCTTGATG

Gene specific 3′-RACE assay primers

Gene	Forward (5′ to 3′ Direction)
BIK1	GCTGGAACACTGCTGAGGTT
KCNMA1	AAGTTACGCCCTCAGAACATTT
NQO1	TGCTCAGAGAAGGAAAAGAC
HMOX1	CTTACACTCAGCTTTCTGGTG
BPNT1	TGGGAGGCAAGTTAACCGAT
VANGL1	CCCTTCCTCTTTCTCCCAGT
FEZ1	CTGGCTACAGCCCTCAAAAA
IGF2	CATGCTTGGGTTTTGTCTTT
AGTR1	TTGCCAGCTATAATCCATCG
RRAS	GGTGTTCGCCATTAACGA
COL5A1	CTGTAATGCCCAGGAAAGGA
Adapter primer	GGCCACGCGTCGACTAGTACTTTTTTTTTTTTTTTTT
AUAP RP	GGCCACGCGTCGACTAGTAC

mRNA half life assay primers

Gene	Forward (5′ to 3′ Direction)	Reverse (5′ to 3′ Direction)
BPNT1	TGGGAGGCAAGTTAACCGAT	GCCCGGCCAAATGAAACTTT
COQ2	TGGTCTTAAGTCAACGGCTCT	GAGTCAGATGGGCTCCTACA
IGF2	TGGTGCTTCTCACCTTCTTG	GAAACAGCACTCCTCAACGA
DNAJC	AGCCAATACGAAGGGGAAGA	GGTGGAAGTGGGAGGAAAGA
GAD1	CTGAGCACACAAACGTCTGT	AATGTCAGACTGGGTAGCGG
GCLC	AAGTTCTTGAAACTCTGCAAGAGAA GG	GCCTCAACTGTATTGAACTCGGAC
GAPDH	GAAGGTCGGAGTCAACGGATTT	GAATTTGCCATGGGTGGAAT
