# Supplementary material for: Star-PAP RNA Binding Landscape Reveals Novel Role of Star-PAP in mRNA Metabolism That Requires RBM10-RNA Association
Source: Int J Mol Sci. 2021 Sep 15;22(18):9980. doi: 10.3390/ijms22189980 (PMC8469156; doi:10.3390/ijms22189980)
Supplement: Supplementary file 1 [file ijms-22-09980-s001.zip › Supplementary Table 3.pdf]

Table S3: List of mRNAs in Star-PAP HITS-CLIP that were undetected after RBM10 depletion

Upregulated mRNAs in siStar PAP microarray

ABHD5	CA5B	DNAJC8	GCA	MAR	PANK1	RARB
ABI1	CA8	DNAL1	GCM1	MARVELD2	PARK2	RBM3
ABR	CABIN1	DPH2	GINS1	MBD1	PBLD	RCN1
ACSM3	CABLES2	DPH5	GLIPR1	MCPH1	PCDH9	RCSD1
ACTR6	CALB1	DRAM2	GOLGA7	MED6	PCOLCE2	RDH11
ADAMTS5	CALU	DZIP1	GON4L	MEF2C	PCSK1	RECK
ADCK1	CASC3	E2F5	GOSR2	METTL3	PDCD10	RELL1
ADIPOR2	CASP7	EBAG9	GPM6A	METTL5	PDCD2	RER1
ADPRHL1	CCDC25	ECHDC1	GPM6B	MGST2	PDE4DIP	RHBDD1
AGO3	CCDC28A	ECSIT	GPR180	MIA3	PDSS2	RNF130
AGPAT5	CCDC53	EFCAB2	GRAMD1A	MINA	PDZRN3	RNF150
AGTR1	CCDC90B	EHHADH	GRM3	MKL1	PEMT	RNF165
AGXT2L2	CCNB1IP1	EIF2S2	GRM5	MKRN2	PHC2	RNF170
AHNAK	CD81	EIF4B	GTF3A	MKX	PHF17	RNF2
AJUBA	CDC42SE2	ELMOD2	GTPBP10	MLLT6	PHF6	RNF217
ALDH8A1	CDCA7	ELSPBP1	GUCY1A3	MPP1	PHF8	RNF5
ANGEL2	CDH11	EMC3	GULP1	MPZL3	PIAS4	RNF6
ANKRD23	CDK6	EML5	HAX1	MRPL45	PLAGL1	RNLS
ANKRD42	CENPQ	ENAH	HBP1	MRPL48	PLEKHM1	RP9
ANKS6	CEP57	EPHA3	HBS1L	MRPS31	PLSCR4	RPE
ANP32B	CHCHD6	ERC2	HDDC2	MSRA	PMF1	RPIA
AP3M1	CHIC1	ERI1	HERPUD2	MTA1	PMPCB	RPP40
APP	CHODL	ETNK2	HPGD	MTAP	PNISR	RQCD1
ARAF	CHRM3	ETV6	HSD17B10	MTHFD2L	PNPLA4	RRAS2
ARHGEF5	CLEC4A	FABP6	HSD17B11	MVB12A	POLR3F	RSL1D1
ASXL1	CLNS1A	FAM105B	HSPA5	MVP	POLR3G	RTN4
ATF7IP	CNIH3	FAM120A	IGBP1	MYC	POLR3GL	RUNX2
ATG4C	CNNM1	FAM122B	IGSF21	MYL6B	POM121C	RUVBL2
ATP6V0A2	CNNM3	FAM20B	IL1RAP	MYLIP	POMZP3	RWDD1
AZI2	CNOT1	FAM92A1	IMMP1L	MYOM1	PON2	SAMSN1
B4GALT5	CNOT4	FANCC	IQCK	N4BP1	POP4	SAR1B
BACE1	COMMD9	FBLN1	ITFG3	NACC1	PPM1K	SCD
BACE2	COMT	FBXL4	KCTD15	NASP	PPP1CB	SCMH1
BAHD1	COPS3	FBXO16	KCTD20	NCAPH2	PPP1R7	SDCBP
BCAP29	COPS7B	FBXO3	KHDC1	NDST1	PPP4R2	SDSL
BCCIP	COQ2	FBXW2	KIAA1239	NDUFA9	PPT1	SEC22A
BCL2L12	COQ5	FCGRT	KIAA1432	NECAP2	PRDM6	SEC22B
BCOR	COX15	FHOD3	KIAA1704	NFATC1	PRICKLE1	SETD7
BCORL1	CPSF6	FILIP1	KLHL12	NHEJ1	PRRG4	SETDB1
BEST1	CREB5	FLCN	KLRC2	NIF3L1	PSMB2	SF3B4
BFAR	CRTAP	FLT1	KLRC3	NLGN1	PSMF1	SGK3
BFSP1	CSNK1A1	FNDC3A	KRBOX4	NLRX1	PTAR1	SGOL1
BPNT1	CSNK1G2	FNTA	KRT222	NOC2L	PTER	SH2B1
BRAP	CTBS	FRK	LAMP2	NRG3	PTP4A1	SH3BP5
BTBD2	CTDSPL	FUCA2	LAMTOR3	NRP2	PTPLAD2	SKI
C11orf74	CTSC	FYN	LANCL2	NRXN3	QKI	SLC12A9
C14orf93	CTSO	FZD3	LAPTM4B	NSMCE2	QRSL1	SLC16A9
C1orf21	CYBB	GAB1	LHPP	NSUN6	RAB10	SLC1A3
C1orf85	CYP27A1	GABPB1	LIX1L	NUB1	RAB12	SLC20A2
C20orf112	CYP2B6	GABPB2	LMAN2L	NUCKS1	RAB22A	SLC24A1
C3orf14	CYP2E1	GAD1	LPXN	NUMA1	RAB5B	SLC2A11
C3orf20	CYP7B1	GALNT10	LRRC34	NUP35	RABL3	SLC35A5
C6orf201	DAG1	GATA3	LRRC7	NUP62CL	RABL6	SLC35F2
C8orf76	DDX50	GATA6	LRRC8B	OCLN	RAD23B	SLC39A14
C9orf41	DERL2	GATM	LRRIQ3	OTUD6B	RANBP17	SLC40A1
C9orf91	DHRS3	GBF1	MAP3K15	OXR1	RAP1GAP2	SLC44A2

SLC7A2	SNAP25	SNX30	SSR1	ST6GALNAC	SUN1	
SMAD4	SNTB1	SP1	SSR4	1	SUV39H1	
SMC3	SNX1	SPG21	SSX2IP	ST7L		
SNAP23	SNX11	SRR	ST6GAL1	STK17A		
TAF8	TBC1D16	TBPL1	TEKT3	TFDP1	TGFBR2	THOC7
TARDBP	TBC1D20	TBX18	TESK2	TFPI	TGFBRAP1	THRB
TATDN2	TBC1D25	TBX19	TFB2M	TGDS	THG1L	
TIMM17B	TMEM184B	TMLHE	TOX	TPMT	TRPC4	TTC23
TJAP1	TMEM189	TMTC1	TPCN2	TRAF6	TRPS1	TTC26
TM9SF1	TMEM27	TMTC2	TPD52	TRAPPC10	TRUB1	TUBD1
TMEM161B	TMEM33	TNFRSF10D	TPD52L1	TRAPPC2	TSPAN18	TXNDC12
TMEM164	TMEM56	TNPO1	TPGS2	TRIM27	TSPAN31	TYRP1
TMEM177	TMEM66	TOR1AIP2	TPK1	TRIM4	TSPAN7	TYW3
U2AF2	UBE2D3	UBE2E3	UBE2H	UBE2W	UBXN2A	UGT8
UNC5A	USP46	USP54	VANGL1	VEGFA	VRK3	WASF2
WDR25	WIPF1	WWOX	YARS	YIPF4	YKT6	ZBTB10
ZBTB16	ZCCHC17	ZDHHC3	ZFAND3	ZFP91	ZFPM2	ZKSCAN3
ZMPSTE24	ZNF226	ZNF330	ZNF780A	ZNF793	ZSCAN25	
ZNF177	ZNF282	ZNF606	ZNF783	ZNRF3		

Upregulated mRNAs in siStar PAP microarray

ABR	GOLGA7	RNF6	ACBD6	ARFGAP3	BMP8A	CCNE2
AGO3	GOSR2	RSL1D1	ACE	ARFIP1	BNIP1	CCNG2
AHNAK	GPM6B	SEC22B	ACO1	ARHGAP5	BNIP3L	CCNT1
AJUBA	GPR180	SH3BP5	ACTN1	ARHGDIB	BOC	CD164
ALDH8A1	HDDC2	SLC1A3	ACTR2	ARHGEF6	BRCC3	CD274
ANGEL2	HERPUD2	SLC20A2	ACTR5	ARL6	BRMS1L	CD44
ANKRD42	LAMP2	SMAD4	ACVR1B	ARMC10	BTBD11	CD97
ANKS6	MAR	SNAP23	ACVR1C	ARRB1	BTF3L4	CDC37L1
ATF7IP	MCPH1	SNTB1	ADAM22	ARRDC4	C10orf54	CDCP1
BACE1	METTL3	SRR	ADAMTS13	ASAH2B	C14orf37	CDH13
BCCIP	MPZL3	STK17A	ADAMTS8	ASB9	C17orf67	CDH23
C20orf112	MTAP	TAF8	ADCY9	ASCC1	C19orf66	CDH3
CALU	MVP	TARDBP	AFAP1L1	ASCC3	C1QL1	CDK1
CASP7	N4BP1	TFDP1	AFG3L2	ASH2L	C21orf58	CDK14
CDC42SE2	NCAPH2	TFPI	AGPAT3	ASTN2	C5orf22	CDK9
CDH11	NECAP2	THOC7	AGRN	ATAD2	C6orf170	CENPK
CHIC1	NFATC1	TMEM161B	AIFM2	ATG16L2	C7orf57	CEP170B
CHRM3	NUCKS1	TMEM189	AK4	ATG4A	C8orf47	CEP250
CNOT1	OCLN	TMEM33	AKAP7	ATL3	C9orf72	CEP55
COX15	OXR1	TNPO1	AKAP9	ATP11B	CAB39	CEP78
CPSF6	PANK1	TPCN2	AKR1C3	ATP13A2	CAB39L	CEP97
CSNK1A1	PCDH9	TPGS2	ALDH2	ATP1B1	CACNB3	CERS5
CYP2E1	PCSK1	TTC23	ALDH7A1	ATP1B3	CAMK2B	CHD5
CYP7B1	PDCD2	UBE2W	AMMECR1	ATP6V1D	CASC4	CHMP7
EIF2S2	PDE4DIP	VRK3	AMMECR1L	ATXN1	CASC5	CHORDC1
ELMOD2	PHF17	ZBTB10	AMN1	ATXN7L1	CASP4	CHST1
FBLN1	PNISR	ZMPSTE24	AMOTL1	AZIN1	CBFA2T2	CHST15
FBXO16	PPP1CB	ZNF226	ANGPT2	BBOX1	CCBL1	CLASP1
FBXO3	PPP1R7	ZNF606	ANKRD1	BCAP31	CCDC113	CLCC1
FHOD3	PPP4R2	AASS	ANKRD12	BEND5	CCDC134	CLEC16A
FILIP1	PSMB2	ABCA7	ANKRD18A	BET1	CCDC150	CLIP2
FLT1	QKI	ABCD3	ANKRD22	BIK	CCDC181	CLMP
FNTA	RAB22A	ABHD12	ANKRD36	BIRC3	CCDC34	CLUAP1
FRK	RABL3	ABHD6	ANTXR1	BLVRA	CCDC57	CLUL1
GALNT10	RANBP17	ACACA	APBB2	BMP6	CCDC69	CNOT7
GATA3	RNF165	ACAD10	ARAP3	BMP7	CCDC82	CNST

COBL	DTX4	FMNL2	HVCN1	LMNB1	MYO5B	PDK1
COL18A1	DYNC2LI1	FNBP1	IER3	LOXL2	MYO6	PDK4
COL4A5	DZANK1	FNDC1	IFI16	LPGAT1	N4BP2L2	PDLIM2
COL5A1	E2F6	FOCAD	IGF2	LRP1	N6AMT1	PDLIM3
COMMD10	EBF2	FOXN2	IGF2BP3	LRP11	NAA40	PDXK
COMMD7	EDEM1	FSD1	IGLON5	LRRC41	NAA60	PDZD4
COPS8	EDNRA	FXYD6	IL11	LSAMP	NAAA	PECR
CORO2A	EEPD1	GAB3	IL12A	LSM14B	NAALAD2	PELI2
CORO6	EIF2S3	GABRE	IL13RA1	LUC7L	NAP1L4	PELI3
CPEB1	EIF3D	GABRQ	IL1A	LYN	NBEAL1	PEX19
CPEB3	EIF3H	GALK1	IL4R	LYPD6B	NCAPD3	PHC1
CPEB4	EIF4E	GALNT6	IMPA1	LYPLA1	NCOA7	PHF20L1
CPOX	EIF4E3	GAS6	INPP5F	LZIC	NDFIP2	PHTF2
CPQ	ELAVL2	GBP1	IQSEC2	MAGED1	NDUFAF7	PIANP
CRIP2	ELF1	GCAT	ISM1	MAGI2	NECAP1	PIGX
CRLF3	ELMO1	GCLM	ITCH	MAN1A2	NEDD9	PIK3CD
CRLS1	ELOVL4	GFPT2	ITGB1BP1	MAN2B1	NEK6	PKIB
CTH	ELOVL7	GFRA1	ITGB3	MAP2	NFYB	PKNOX2
CTNNBIP1	ENO1	GHR	ITGB5	MAP2K1	NGEF	PLD6
CTSS	ENOX2	GK5	ITGB8	MAP3K7CL	NID1	PLEK2
CTTNBP2NL	ENTPD1	GLCCI1	ITPR2	MAP3K8	NLRC5	PLEKHA2
CUL5	EOGT	GLTSCR1L	ITSN1	MAP4K4	NOL4	PLEKHB2
CUL9	EPHX2	GMFB	JAG2	MAPK14	NPHP3	PLEKHG1
CUX2	EPS8L2	GNAI1	JAM2	MAPRE2	NR1D2	PLEKHG4B
CXADR	ERBB3	GNB1L	JKAMP	MAPRE3	NR5A2	PLIN3
CXorf38	ERCC3	GNG2	JMY	MAST3	NRIP3	PNPLA8
CXorf57	ESCO2	GOPC	JPH1	MAT2B	NUAK1	PODXL
CYB5R2	ESYT2	GPC3	JUP	MATR3	NUSAP1	POLH
CYB5RL	ETHE1	GPLD1	KAT2B	MBP	NXPE3	PPAPDC1B
CYP27C1	ETS1	GPNMB	KAZN	MBTPS2	OBFC1	PPARA
CYP2S1	EVA1C	GPR137B	KCNJ4	MCAM	OBSCN	PPEF1
DACT3	EWSR1	GPR137C	KCNK12	MCTP2	OBSL1	PPFIA4
DCAF12	EXOC5	GPR143	KCNK2	MDM2	OCIAD1	PPHLN1
DCAF5	EXOG	GRB2	KCTD13	MEAF6	OGFRL1	PPID
DCK	EXTL2	GREB1	KDELC2	MED27	OSBPL10	PPM1H
DCUN1D1	FADS1	GRID1	KDM4C	MEGF9	OSGIN2	PPP1R12B
DDAH1	FAM105A	GSTO2	KIAA1107	MFAP3L	OTUD3	PPP2CA
DDHD1	FAM134B	GTPBP4	KIAA1147	MGA	P2RX4	PPP2R2C
DDR1	FAM161A	GUCY1A2	KIAA1244	MGST3	PAFAH1B1	PPP2R3A
DDX17	FAM171B	GXYLT2	KIAA1551	MICU2	PAG1	PPP3CB
DDX18	FAM175A	GZF1	KIF16B	MID1	PAK1	PPP3R1
DEAF1	FAM177A1	HABP4	KIF3A	MITF	PALM2	PPP6C
DENND1C	FAM184A	HAUS2	KIF3C	MLF1	PALMD	PRDM2
DERL1	FAM214A	HIVEP1	KLF12	MLKL	PANK2	PRDX1
DGCR2	FAM228B	HK1	KLHL13	MMP13	PANK3	PRELID2
DHDDS	FAM63B	HLA-A	KRAS	MMP28	PAPOLA	PRICKLE2
DHRS1	FAM64A	HLA-C	KREMEN1	MOB1A	PAPPA	PRKACA
DHRS9	FAM98B	HLA-F	KRT80	MOB4	PAQR5	PRKACB
DIAPH1	FAR1	HLA-G	LAMB1	MPHOSPH8	PBK	PRKAR2B
DIDO1	FASN	HMGN3	LAMP1	MPZL2	PBX1	PRKDC
DIP2B	FBLN5	HMOX1	LAMP5	MSRB3	PBX3	PRKRIP1
DKK3	FBXL13	HNRNPD	LAP3	MTFMT	PCBP4	PRR5
DLD	FBXL21	HOMER3	LARP1B	MTMR7	PCF11	PRR5L
DNAJB6	FBXL5	HP1BP3	LARS	MTPAP	PCID2	PRRC1
DNAJC18	FBXO2	HSPA14	LATS2	MTUS1	PCOLCE	PRSS27
DNAJC27	FBXO22	HSPB11	LCOR	MTX3	PCYOX1	PSD3
DNM1L	FBXO32	HTR3A	LDLRAP1	MYBPC2	PDCD6	PSMA5
DOCK4	FDFT1	HTRA1	LETM2	MYH9	PDGFC	PTBP3
DOCK6	FEZ1	HUS1	LIG3	MYO1D	PDGFD	PTGFRN
DOK5	FLVCR2	HUWE1	LIN7A	MYO3A	PDGFRL	PTPRG

PUS7L	RIC8B	RTN1	SFT2D2	SLC38A2	STK4	TAPT1
PVR	RIMKLB	RTN3	SGK1	SLC39A9	STX16	TBC1D7
RAB2A	RIMS2	RUNX1T1	SGMS2	SNAPC3	STX6	TBC1D8B
RAB2B	RIMS3	RWDD4	SGSM3	SNTA1	STYX	TBCEL
RAB6B	RLF	RYR1	SH3GL2	SNX29	SULF2	TBX1
RAB8B	RMND5A	RYR2	SHC3	SOGA3	SULT1A1	TCF12
RABEPK	RNASEH2B	SAMD14	SHC4	SOX5	SULT1C2	TCF7L1
RAC2	RNF115	SAMD8	SHE	SOX7	SULT4A1	TCFL5
RAD51B	RNF14	SAR1A	SHOC2	SP4	SUMF2	TEX261
RALGDS	RNF141	SBNO1	SLAIN1	SPATA18	SUV39H2	TFEB
RAP1A	RNF144A	SCARA3	SLAIN2	SPG20	SWAP70	TGFA
RAPGEF6	RNF157	SCML1	SLC12A8	SPRED2	SYNE1	TGFBR1
RASSF2	RNF169	SCRN3	SLC16A7	SPTY2D1	SYNE2	TGM2
RB1	RNF175	SDE2	SLC22A15	SRPK2	SYNGAP1	TIAM1
RBFOX2	RNPEP	SDR16C5	SLC25A15	SRPRB	SYNRG	TM2D1
RBM33	ROR1	SEC22C	SLC25A16	SRSF4	SYT11	TMEM106B
RCHY1	RPRD1A	SEC62	SLC25A46	ST6GAL2	SYT13	TMEM117
RDH10	RPS6KA1	SEMA4D	SLC30A10	ST6GALNAC	SYT17	TMEM130
REEP1	RPS6KA2	SEPT	SLC30A5	2	SYT9	TMEM154
RETSAT	RRAGD	SERBP1	SLC33A1	ST8SIA5	SYTL2	TMEM155
REXO2	RRM2B	SERPINB2	SLC35A3	STARD10	T TSPAN2	TMEM163
RFC3	RSAD1	SERPINC1	SLC35B3	STARD7	TTC19	ZMYND8
RFX7	RSBN1	SERPINF1	SLC35C2	STC1	TTL	ZNF148
RGS4	RSPH3	SESTD1	SLC35F3	STK17B	TACC1	ZRANB1
RHBDL3	RSU1	SFRP4	SLC38A10	STK3	TAF9B	ZSCAN20
TMEM182	TMEM38A	TMEM65	TOLLIP	TRIM22	TSFM	
TMEM206	TMEM38B	TMEM68	TOMM34	TRIM25	TSNAX	
TMEM237	TMEM55A	TNFRSF21	TRABD2B	TRIM58	TSPAN11	
TMEM30A	TMEM63A	TNFRSF9	TRAPPC9	TRIM6	TSPAN14	
TTLL1	TXNDC9	UBE2L6	UCK2	ULK2	USP12	
TTYH2	UBE2B	UBE3C	UGCG	UNC13B	USP34	
TWF1	UBE2D2	UBE4B	UGT3A1	URI1	USP9X	
TXLNB	UBE2K	UBXN6	UHMK1	USP1	UTP20	
VARS2	VDAC3	VSTM4	WDR31	WIF1	WWP2	
VASH2	VNN2	VTI1A	WDR34	WNT2B	XRCC6BP1	
VAT1L	VPS41	WARS	WDR44	WTAP	YAP1	
YIPF6	ZBTB44	ZC3H12A	ZDHHC11	ZFP28	ZKSCAN8	
YWHAZ	ZC2HC1A	ZDHHC1	ZFAND4	ZFYVE28	ZMAT3	
ZNF180	ZNF37A	ZNF510	ZNF618	ZNF704		
ZNF25	ZNF397	ZNF557	ZNF641	ZNF75D		
ZNF283	ZNF483	ZNF589	ZNF658	ZNF846		

mRNAs not overlapped with siStar-PAP microarray

AAED1	ACTC1	ADH5	AGMAT	AKR1C2	AMACR	ANKAR
AASDHPPT	ACTL6B	ADH6	AGR2	AKR1C4	AMBP	ANKDD1A
AB231702	ACTR3C	ADH7	AGR3	AL137655	AMDHD1	ANKEF1
AB231731	ACTR8	ADHFE1	AGTR2	AL832184	AMELX	ANKRA2
ABCA1	ADAM18	ADORA3	AIFM1	ALAS2	AMICA1	ANKRD2
ABCA13	ADAMTS4	AEBP2	AIM2	ALDH1A1	AMTN	ANKRD32
ABHD10	ADAMTSL3	AFTPH	AIMP1	ALDH5A1	ANAPC10	ANKRD35
ABO	ADARB2	AGAP7	AJ606316	ALDOB	ANAPC5	ANKRD36B
ACADS	ADAT2	AGAP8	AJ606331	ALG13	ANGPT1	ANKRD45
ACAT1	ADD1	AGAP9	AJAP1	ALG1L	ANGPTL1	ANKRD46
ACBD3	ADH1A	AGBL2	AK1	ALG1L2	ANGPTL3	ANKRD7
ACBD7	ADH1C	AGFG2	AK131325	ALKBH8	ANGPTL5	ANKUB1
ACSM2A	ADH4	AGGF1	AK5	ALS2CR8	ANHX	AP1AR

AP3M2	BC038536	C16orf97	CASP3	CD8A	CLSTN1	CXorf48
APEX2	BC039000	C17orf70	CASP5	CD8B	CLTA	CXorf56
APIP	BC041470	C17orf85	CASP6	CD96	CLVS2	CXorf58
APLF	BC043280	C18orf54	CASP8AP2	CDC20	CMAS	CYLC2
APMAP	BC043355	C19orf45	CASP9	CDH24	CNGA2	CYP2A7
APOO	BC043529	C1orf123	CASQ1	CDIP1	CNGA3	CYP2C18
APOOL	BC043546	C1orf146	CASR	CDK11A	CNKSR2	CYP2J2
AQP1	BC071797	C1orf186	CASS4	CDK12	CNN3	CYP46A1
AQP9	BC073807	C1orf43	CATSPER2	CDK15	CNNM4	CYP4B1
ARF5	BCAS1	C1orf61	CATSPER3	CDKAL1	CNOT11	CYTH1
ARG1	BCL2L11	C1orf63	CAV2	CDKL1	CNOT8	CYTH3
ARG2	BCL2L13	C1orf94	CBWD1	CDKL4	CNTN6	CYTIP
ARHGAP15	BCL2L14	C2	CBX3	CDKN3	CNTNAP3	DAB2IP
ARHGAP20	BCL6	C2orf43	CBY1	CDON	COG3	DAGLB
ARHGAP22	BCO2	C2orf61	CCDC108	CEACAM4	COL15A1	DAOA
ARHGAP25	BDH1	C2orf76	CCDC11	CELA2B	COL23A1	DBC1
ARHGEF11	BEND7	C2orf80	CCDC111	CELA3A	COL26A1	DBF4B
ARHGEF17	BGN	C3orf17	CCDC112	CELF2	COL2A1	DBR1
ARL1	BHMT2	C3orf37	CCDC129	CELF3	COL6A3	DCAF10
ARL5A	BIRC2	C4B_2	CCDC14	CENPO	COLEC10	DCAF13
ARL8A	BLK	C4BPB	CCDC149	CENPP	COMMD5	DCBLD1
ARL8B	BLOC1S5	C4orf17	CCDC152	CEP104	COPE	DCLRE1A
ARMC1	BMP5	C4orf22	CCDC169	CEP57L1	COPS4	DCPS
ARMC12	BMPR1B	C4orf27	CCDC171	CERS1	COQ3	DCST2
ARMC6	BOP1	C4orf40	CCDC172	CFC1	COX18	DCTN3
ARMCX5-	BPIFA2	C4orf47	CCDC173	CGREF1	CPA2	DCTN6
GPRASP2	BPIFA3	C5orf45	CCDC22	CHCHD3	CPAMD8	DCUN1D2
ARRDC3	BPIFB1	C6orf10	CCDC6	CHD6	CPE	DCUN1D5
ARSB	BRCA2	C6orf118	CCDC64	CHI3L1	CPM	DCX
ASB11	BSPRY	C6orf185	CCDC85C	CHMP3	CPN1	DDI2
ASB5	BTBD1	C6orf58	CCL25	CHMP5	CPNE3	DDRGK1
ASIC3	BTBD10	C7orf25	CCNB1	CHN1	CPNE8	DDX24
ASPN	BTBD7	C7orf26	CCND1	CHPT1	CPO	DDX43
ASRGL1	BTBD8	C7orf63	CCNDBP1	CHRDL2	CPSF4	DDX59
ASTE1	BTBD9	C8orf31	CCNG1	CHRFAM7A	CR933660	DEC
ASUN	BTG4	C8orf37	CCNI	CHRNA2	CR936796	DENR
ATAD1	BTK	C8orf46	CCNJ	CHRNB3	CRBN	DEPDC4
ATF1	BTNL3	C9orf135	CCNJL	CHST10	CREB1	DEPTOR
ATG14	BTNL8	C9orf171	CCNL1	CHUK	CREB3L1	DERA
ATG5	BVES	C9orf24	CCS	CHURC1-	CRISP1	DES
ATP2B2	BX537783	C9orf78	CCT6A	FNTB	CRISP2	DESI1
ATP2B3	BX647938	C9orf89	CD160	CIAPIN1	CRISP3	DFFA
ATP4B	C10orf107	CA1	CD200R1	CIB4	CROCC	DFNB59
ATP5F1	C10orf11	CA13	CD200R1L	CILP	CRYL1	DGAT2
ATP5J2-	C10orf128	CA2	CD207	CKM	CSF1	DGAT2L6
PTCD1	C10orf131	CA3	CD244	CKMT1B	CSPG4	DGKB
ATP5SL	C10orf32	CA6	CD247	CLEC10A	CSPP1	DGKD
ATP6V0D2	C10orf88	CA7	CD33	CLEC12A	CSRNP3	DGKK
ATP6V1B1	C10orf90	CAAP1	CD37	CLEC1A	CSRP2	DGUOK
AURKA	C11orf48	CABP4	CD38	CLEC1B	CSRP2BP	DHDH
AWAT1	C11orf65	CACNA1E	CD53	CLEC2D	CSRP3	DHRS12
AXIN2	C11orf82	CACNA1S	CD55	CLEC4C	CTDNEP1	DHRS7
AY769439	C11orf84	CACNG5	CD58	CLEC4D	CTSE	DHX30
B4GALT4	C11orf88	CACYBP	CD5L	CLEC6A	CTSH	DHX32
BACH2	C12orf29	CALCOCO1	CD6	CLEC7A	CTSK	DHX33
BAI1	C12orf56	CALN1	CD74	CLEC9A	CUL1	DIRC2
BC015433	C14orf105	CALR3	CD80	CLIC2	CUTC	DKFZp434C06
BC022056	C14orf166	CAPG	CD82	CLIC4	CWC15	31
BC035867	C15orf43	CASD1	CD84	CLIC6	CWC25	DKFZp434F16
BC038532	C16orf89	CASP12	CD86	CLN3	CXorf30	22

DNAH14	ENAM	FAM214B	FZD6	GORASP2	HEATR5B	IL17RA
DNAJA1	ENDOU	FAM219A	G2E3	GOT1	HEG1	IL19
DNAJA2	ENPP6	FAM221A	GABRA2	GP6	HENMT1	IL1RL1
DNAJB11	ENTHD1	FAM228A	GABRA4	GPAT2	HEPACAM2	IL20RB
DNAJC5B	ENTPD5	FAM35A	GABRB2	GPC4	HERC3	IL22RA2
DNALI1	EP400	FAM47E	GABRG1	GPC5	HEXB	IL24
DNASE1L3	EPB41L3	FAM47E-	GABRR2	GPD1L	HEXDC	IL2RA
DNMBP	EPB42	STBD1	GAGE8	GPHN	HGD	IL33
DNTTIP2	EPCAM	FAM5B	GALM	GPN3	HHIPL1	IL36B
DOK2	EPHA10	FAM5C	GALNT8	GPR111	HHIPL2	IL6
DOK6	EPHX4	FAM71E2	GAPVD1	GPR115	HHLA1	IL6R
DONSON	EPYC	FAM86B2	GAR1	GPR144	HIBADH	IL7
DPF3	EQTN	FANCD2	GAS2	GPR153	HID1	ILDR2
DPH6	ERAL1	FARS2	GATA4	GPR156	HIF3A	IMMP2L
DPM1	ERBB2	FASTKD3	GATSL1	GPR161	HJURP	IMMT
DPPA2	ERCC2	FBL	GBGT1	GPR89C	HK3	INCA1
DPPA4	ERCC5	FBLN7	GCH1	GPRC6A	HKR1	ING5
DPY19L1P2	ERCC6L2	FBN3	GCK	GRAP2	HLA-DRB5	INPP1
DPY19L2	ERI3	FBP1	GCNT7	GRIA4	HMBOX1	INPP5J
DPY19L3	ERLIN1	FBXO31	GCSAML	GRID2	HMGCL	INSIG2
DRC1	ERLIN2	FCER1A	GDF1	GRIK2	HMGN5	INTS12
DRD2	ERMAP	FCF1	GDI2	GRIK5	HNRNPAB	INTS8
DRD3	ERP27	FCGR2B	GDPD1	GRIN2D	HNRNPC	IP6K1
DRG1	ERP44	FCN3	GEMIN2	GRK1	HNRNPH3	IP6K3
DSCC1	ESPN	FCRL1	GFI1B	GSAP	HNRNPL	IPMK
DTX1	ESRRG	FER1L5	GFRA2	GSE1	HOMER1	IQCA1L
DUSP11	EU233817	FERMT1	GFRA3	GSS	HP11113	IRAK3
DVL1	EXD3	FETUB	GFRAL	GSTA1	HPRT1	IREB2
DYX1C1	EXOC4	FEZ2	GGH	GSTA2	HR	IRF2
E2F2	EXOSC1	FGB	GHITM	GSTA3	HRG	IRF6
ECSCR	EXOSC8	FGFR4	GIF	GSTA5	HRSP12	ISM2
EDA2R	EXTL3	FGG	GIMAP1-	GSTM2	HS1BP3	ITGA10
EDARADD	EZH2	FGGY	GIMAP5	GSTM3	HSD11B1	ITGAL
EDNRB	F10	FGL1	GIN1	GSTO1	HSD17B13	ITLN1
EEFSEC	F11R	FH	GIPC1	GTDC1	HSD17B3	ITLN2
EF070117	F3	FHL2	GKN1	GTF2A1L	HSD17B7	ITM2A
EF070119	F9	FHL5	GKN2	GTF2B	HSF2	IYD
EFCAB12	FADS6	FIGF	GLIS1	GTF2E2	HSH2D	IZUMO1
EFCAB5	FAHD2B	FKBP2	GLIS3	GTF2F2	HSPA12B	JAK2
EFCAB6	FAM102B	FKBP3	GLO1	GTF2H2C	HSPE1-MOB4	JARID2
EFCC1	FAM115A	FKBP6	GLRA1	GTF3C5	HTN3	JHDM1D
EFTUD2	FAM115C	FLI1	GLT1D1	GTF3C6	HTR2C	KANSL2
EGFR	FAM117A	FLJ00385	GLYAT	GTSF1	HTR3B	KAT6A
EHF	FAM118B	FLJ22184	GLYATL1	GUCA1A	HTR3C	KCNC2
EIF1AX	FAM120B	FLOT2	GLYATL3	GUK1	HTR3E	KCND2
EIF2S1	FAM135B	FMO1	GMNN	GXYLT1	HTR4	KCND3
EIF3B	FAM157A	FMO2	GMPR	H2AFY2	HYAL1	KCNH1
EIF3J	FAM157B	FOXN1	GNA14	HAAO	IBSP	KCNH8
EIF3K	FAM161B	FOXN4	GNAI3	HABP2	IFFO2	KCNIP1
EIF4ENIF1	FAM163A	FOXP4	GNAO1	HAO1	IFI44L	KCNIP3
ELAVL4	FAM168A	FOXR1	GNAT3	HAO2	IFNAR2	KCNIP4
ELMOD3	FAM168B	FRMD1	GNE	HAP1	IFNGR1	KCNK10
ELMSAN1	FAM171A1	FRMD7	GNL3L	HAT1	IFNLR1	KCNN1
ELOVL2	FAM178A	FRMPD1	GNPAT	HCCS	IFT52	KCNN2
ELOVL5	FAM178B	FRMPD2	GNPDA1	HCN1	IFT57	KCNN3
EMC1	FAM185A	FRS2	GNPDA2	HCN4	IGFBP3	KCNN4
EMC2	FAM194B	FSTL4	GOLGA6B	HCRTR2	IGHMBP2	KCNQ1
EMD	FAM203A	FTO	GOLGA6L1	HDAC3	IGSF11	KCTD18
EME1	FAM206A	FUCA1	GOLGA6L10	HDAC6	IL13RA2	KCTD19
EML1	FAM208A	FUT8	GOLGA6L9	HDAC7	IL15RA	KDM5D

KHDRBS2
KHDRBS3
KIAA0146
KIAA0895
KIAA1045
KIAA1210
KIAA1257
KIAA1279
KIAA1614
KIAA1751
KIAA1804
KIF17
KIF27
KIR2DL1
KIR2DL2
KIR2DL3
KIR2DL4
KIR2DL5B
KIR2DS1
KIR2DS2
KIR2DS5
KIR3DL1
KIR3DS1
KLHDC8B
KLHL1
KLHL14
KLHL18
KLHL22
KLHL32
KLHL6
KLK9
KLRB1
KLRC1
KLRD1
KLRF1
KLRF2
KLRK1
KNSTRN
KRT10
KRT19
KRT20
KRT23
KRT24
KRT25
KRT27
KRT33B
KRT39
KRT40
KRT6A
KRT6B
KRT6C
KRT71
KRT77
KSR1
KY
LACE1
LACTB2
LAPTM4A
LBP
LCA5
LCLAT1
LCN1
LCN8
LDB1
LDB2
LDHA
LDHAL6A
LDHB
LDHC
LDLRAD3
LECT1
LEMD1
LGALS9B
LGI1
LGI2
LGR5
LILRA2
LILRA4
LINGO2
LIPC
LIPJ
LIPK
LIPN
LMAN1L
LOC100127983
LOC100129636
LOC100132247
LOC100287534
LOC100288332
LOC100506422
LOC100996485
LOC388813
LOC440243
LOC642778
LPHN2
LPPR1
LPPR4
LPPR5
LRP2BP
LRRC16A
LRRC2
LRRC23
LRRC27
LRRC31
LRRC39
LRRC42
LRRC72
LSM14A
LUC7L3
LXN
LYG1
LYG2
M1AP
MACC1
MAOA
MAP2K7
MAP3K14
MAP3K4
MAPK1
MAPK13
MAPRE1
MAPT
MAST1
MATK
MATN2
MATN3
MAVS
MBD4
MBIP
MBNL2
MBNL3
MCHR2
MCIDAS
MCM3
MDH1
MDM4
MED12
MED24
MED25
MEF2B
MEF2BNB-MEF2B
MEP1B
MFAP1
MFAP4
MFAP5
MFSD6
MFSD9
MGAT4C
MGAT5B
MICALCL
MICU1
MID2
MINK1
MITD1
MLC1
MLF2
MLXIP
MMADHC
MMP11
MMP26
MMP7
MMP8
MMRN1
MMRN2
MMS19
MNDA
MNS1
MOCS2
MOV10L1
MPDZ
MPPED1
MPPED2
MPV17
MRPL13
MRPL21
MRPL22
MRPL24
MRPL47
MRPS10
MRPS18A
MRPS22
MRPS27
MRPS35
MS4A1
MS4A13
MS4A3
MS4A4A
MS4A6A
MSI1
MSL3
MSS51
MST1L
MTDH
MTERFD1
MTFR1
MTMR14
MTR
MTRF1L
MTSS1
MTTP
MUC6
MXD1
MXRA5
MYBPHL
MYL1
MYL2
MYL3
MYO7B
MYOZ1
MYSM1
MYT1L
MZF1
NAA20
NADKD1
NAF1
NAGA
NAPEPLD
NBPF14
NCAM1
NCAPH
NCDN
NCF1
NCF2
NCOR1
NCR2
NDUFV2
NEDD8-MDP1
NEGR1
NEK9
NEURL
NFIX
NHLRC3
NHSL2
NIPAL1
NIPAL2
NIPAL4
NIPSNAP3B
NKAIN3
NKAP
NKD1
NKD2
NLE1
NLGN4X
NLGN4Y
NLN
NLRP11
NLRP12
NLRP3
NLRP4
NLRP8
NLRP9
NME5
NME9
NMI
NMS
NMU
NOA1
NOB1
NOD1
NOM1
NOP58
NOS1AP
NPC1L1
NPHS2
NPIP
NPL
NPLOC4
NPR1
NPR3
NPSR1
NPTN
NR1I2
NR1I3
NR2E3
NR3C1
NR3C2
NR4A3
NSDHL
NSFL1C
NSL1
NT5C1B
NTM
NTN5
NUDC
NUDCD3
NUDT12
NUDT13
NUDT5
NUDT9
NUMB
NUP205
NUP37
NUPL2
NUTM1
NXF3
NXPE2
NXPE4
OARD1
OBP2B
OCIAD2
ODAM
OGDH
OGN
OLAH
OLFM3
OLFML2B
OMA1
OPALIN
OPCML
OPN1LW
OPN1MW
OPN5
OPTC
ORC6
OSMR
OSTM1
OTC
OTOF
OTOP2
OTOP3
PACRG
PACS1
PADI2
PALM2-AKAP2
PAPL
PARK7
PARP11
PARP12
PATL2
PAX2
PAXIP1
PBXIP1
PCDH11X
PCDH19
PCGF2
PCM1
PCMT1
PCSK6
PCYOX1L
PCYT1B
PDCD1LG2
PDLIM1
PDPN
PEAK1
PEBP4
PEG3
PEX3
PGAP2
PGBD1
PGGT1B
PGLYRP3
PGLYRP4
PHKB
PHKG1
PI15
PIGF
PIGG
PIGR
PIGS
PIH1D1
PIH1D2
PIH1D3
PIK3AP1
PILRA
PINLYP
PINX1
PIP4K2A
PIWIL4
PKD2L2
PLA2G4F
PLA2G5
PLEK
PLEKHA6
PLEKHB1
PLEKHD1
PLEKHG5
PLEKHM3
PLGRKT
PLSCR1
PLSCR2
PLSCR5
PLXNA2
PLXNB3
PMFBP1
PMPCA
PMS2L14
PNKD
PNLDC1
PNLIPRP2
PNO1
PNPLA1
POC1A
PODXL2
POFUT1
POGK
POLA2
POLDIP3
POLE2
POLR2J2
POLR2J3
POLR3E
POMP
POMT1
PON1
PON3
POTEE
POTEI
POTEJ
POU1F1
PPAP2B
PPAPDC1A

PPARGC1B	PTPN23	RGSL1	SAFB	SLC15A4	SNRNP48	STPG2
PPFIA3	PTPRF	RHAG	SAMD7	SLC15A5	SNRNP70	STRA8
PPIL6	PTPRO	RHCE	SARDH	SLC16A4	SNRPB2	STT3A
PPM1F	PTS	RHD	SART1	SLC17A6	SNRPC	STX12
PPP1R13B	PTTG1	RHEB	SCAMP5	SLC19A1	SNTG2	STX8
PPP1R13L	PUM2	RHPN1	SCARA5	SLC19A2	SNURF-	STXBP1
PPP1R36	PVRL1	RIIAD1	SCFD2	SLC1A4	SNRPN	SUCLG1
PPP1R42	PYCRL	RILP	SCHIP1	SLC1A5	SNX16	SUGP1
PPP2CB	QDPR	RILPL1	SCPEP1	SLC22A25	SNX31	SULT1B1
PPP3CC	QPCT	RIOK1	SCYL3	SLC22A9	SNX7	SULT1C3
PPP6R1	QSOX1	RIPK4	SDCCAG3	SLC24A5	SOD2	SULT1E1
PPP6R3	R3HCC1	RIT1	SDHB	SLC25A14	SOX13	SULT2B1
PPTC7	RAB14	RMDN2	SDK2	SLC25A17	SP140	SULT6B1
PQLC3	RAB15	RNF103-	SEC31A	SLC25A20	SPACA1	SUN3
PRAP1	RAB18	CHMP3	SECISBP2	SLC25A27	SPACA7	SUPT5H
PRDM1	RAB21	RNF125	SEH1L	SLC25A33	SPAG8	SV2B
PRDM14	RAB23	RNF128	SERAC1	SLC25A42	SPARCL1	SVOPL
PRDM7	RAB26	RNF138	SERPINA3	SLC26A3	SPATA22	SYCP2L
PRDX4	RAB27A	RNF144B	SERPINB10	SLC27A4	SPATA31C1	SYCP3
PRIM2	RAB28	RNF149	SERPINB11	SLC27A5	SPATA9	SYDE2
PRKAB1	RAB4A	RNF185	SERPINB12	SLC2A1	SPDEF	SYF2
PRKAB2	RAB6A	RNF32	SERPINB3	SLC2A2	SPDYE1	SYNE3
PRKAG1	RABL2B	RNF8	SERPINB7	SLC2A5	SPDYE5	SYT10
PRKCG	RAC1	RNFT1	SERPINE3	SLC30A4	SPDYE6	SYT2
PRKX	RAD23A	RNPS1	SERPINI1	SLC34A1	SPG11	SYT3
PROC	RAD51AP1	ROPN1	SERPINI2	SLC35E2B	SPIC	SYTL3
PROSC	RAD51C	RPA2	SETD2	SLC35F1	SPIN1	TAB2
PROZ	RADIL	RPA3	SETDB2	SLC36A3	SPIRE2	TAF3
PRPF3	RALYL	RPF1	SF3B2	SLC38A11	SPON2	TAF4
PRPF8	RAPGEF3	RPF2	SFSWAP	SLC38A5	SPP2	TAMM41
PRPS2	RAPSN	RPH3AL	SFTPD	SLC38A7	SPPL2B	TAOK3
PRR14L	RARA	RPN2	SGCD	SLC38A8	SRFBP1	TARP
PRRC2B	RARG	RPRD1B	SGCG	SLC41A1	SRGAP2C	TAS1R1
PRRG2	RB1CC1	RPS10-	SGCZ	SLC45A2	SRGAP2D	TAS1R2
PRSS3	RBAK	NUDT3	SGK2	SLC45A4	SRI	TAZ
PRSS45	RBAK-	RPS20	SGMS1	SLC5A7	SRMS	TBATA
PRSS54	RBAKDN	RPS4X	SGSM1	SLC5A9	SRPX	TBC1D15
PRUNE	RBKS	RPS4Y1	SGSM2	SLC6A11	SRSF5	TBC1D3B
PSAP	RBL1	RPS6KB2	SH2B2	SLC6A14	SSBP1	TBC1D3C
PSAT1	RBM10	RPTOR	SH2D3A	SLC6A8	SSPO	TBC1D5
PSG3	RBM41	RRAGC	SH2D4B	SLC7A14	SSUH2	TBCCD1
PSG6	RBM45	RRH	SHANK1	SLC7A9	SSX3	TBP
PSMA2	RBMX2	RRP12	SHCBP1L	SLCO1B3	SSX4B	TBPL2
PSMA4	RBMY1F	RRP1B	SIGLEC12	SLCO5A1	SSX7	TBX20
PSMA6	RBPMS2	RS1	SIGLEC5	SLMO1	ST18	TBX22
PSMB1	RBSG1	RSBN1L	SIGLEC6	SLX4IP	ST3GAL4	TBX4
PSMB7	RCL1	RSPH1	SIGLECL1	SMAD3	ST6GALNAC	TBX5
PSMD11	RCN2	RSPH10B	SIN3B	SMAP1	6	TCEB1
PSMD12	RCOR3	RSPH4A	SIRT1	SMAP2	ST8SIA6	TCF7L2
PSME4	RDH12	RSRC1	SIRT3	SMARCD3	STAC2	TCN1
PSPH	REG1B	RTCA	SKA3	SMC4	STAP1	TCN2
PTBP2	REG4	RTDR1	SKIL	SMCO2	STAR	TCP10
PTCD2	REP15	RTFDC1	SKP2	SMNDC1	STARD3NL	TCP10L2
PTCH2	RFESD	RTKN2	SLA	SMOX	STATH	TCP11
PTCHD2	RFTN1	RTN4IP1	SLA2	SMPD1	STEAP4	TCP11L2
PTEN	RFTN2	RXRG	SLAMF1	SMPDL3A	STK16	TCP11X2
PTK7	RGN	S100PBP	SLAMF6	SMPDL3B	STK32A	TDRD10
PTPLA	RGR	SAA2-SAA4	SLAMF7	SMYD2	STMN2	TDRD7
PTPN20A	RGS20	SACS	SLBP	SMYD3	STOML3	TEAD3
PTPN20B	RGS8	SAE1	SLC15A3	SNRK	STPG1	TEKT1

TELO2	TMEM110	TPST2	TSPAN33	UGT2B28	VSTM1	XYLT2
TEP1	TMEM132C	TPTE2	TSPAN5	UGT3A2	VTA1	YBX1
TEPP	TMEM132E	TRA2A	TSPAN8	UMOD	VTCN1	YTHDF2
TEX101	TMEM143	TRABD2A	TSR1	UNC80	VWDE	YTHDF3
TEX29	TMEM14B	TRAPPC6A	TSSC1	UPB1	WARS2	YWHAB
TEX33	TMEM150B	TREML4	TTC1	UPF3A	WASH1	YY1AP1
TFAP2B	TMEM150C	TRIM29	TTC18	UPK1B	WASH7P	ZBTB17
TFAP2D	TMEM156	TRIM34	TTC21A	UPK3B	WBSCR16	ZBTB38
TFAP2E	TMEM184C	TRIM35	TTC39A	UPP2	WDFY2	ZBTB49
TFB1M	TMEM189-	TRIM38	TTC8	URGCP-	WDHD1	ZC2HC1B
TFE3	UBE2V1	TRIM43	TTI1	MRPS24	WDPCP	ZC3H12C
TFEC	TMEM194B	TRIM48	TTLL10	USF2	WDR19	ZC3H7B
TFG	TMEM218	TRIM49	TTLL11	USHBP1	WDR5	ZC3H8
TFR2	TMEM220	TRIM49B	TTLL12	USP18	WDR59	ZC3HC1
TG	TMEM232	TRIM50	TTLL6	USP26	WDR62	ZCCHC14
TGFB3	TMEM248	TRIM51	TULP4	USP3	WDR82	ZCCHC9
THEGL	TMEM25	TRIM63	TVP23A	USP38	WDR86	ZCRB1
THEM4	TMEM253	TRIM64B	TXNDC8	USP49	WDYHV1	ZDHHC14
THEM5	TMEM254	TRIM77	TXNRD2	USP8	WEE2	ZDHHC17
THEMIS	TMEM45B	TRIML1	UBA2	UST	WFDC1	ZDHHC18
THOC1	TMEM56-	TRIT1	UBA7	UTS2	WFDC3	ZDHHC19
THOC3	RWDD3	TRMT10A	UBAC2	UTS2B	WFDC8	ZFAND1
THOP1	TMEM57	TRMT10B	UBAP1L	UTY	WHAMM	ZFAND6
THSD7B	TMEM71	TRMT44	UBE2A	VBP1	WIPF3	ZFAT
TIMM17A	TMEM87B	TRMT61B	UBE2J1	VCAM1	WIZ	ZFP69
TIMM23	TMX4	TRMU	UBE2T	VCAN	WNK3	ZFR2
TIMMDC1	TNFSF12	TROVE2	UBE2U	VIL1	WNT8A	ZFYVE1
TLDC1	TNIP2	TRPA1	UBXN11	VIM	WSB2	ZFYVE21
TLE1	TNNI1	TRPC4AP	UBXN2B	VIP	WSCD1	ZGPAT
TMA16	TNNT2	TRPC5	UBXN8	VNN1	WSCD2	ZHX1-
TMBIM4	TOE1	TRPV6	UCHL1	VPS26A	XG	C8ORF76
TMC4	TOM1L2	TSG101	UCHL3	VPS39	XKR5	ZKSCAN2
TMC7	TOX4	TSGA13	UCP1	VSIG1	XPNPEP2	ZKSCAN5
TMCO1	TP53BP2	TSHR	UCP3	VSIG10	XPOT	ZKSCAN7
TMCO3	TPD52L2	TSPAN13	UGT2B10	VSIG4	XRCC4	ZMAT4
TMED8	TPM4	TSPAN19	UGT2B11	VSIG8	XRCC6	ZMAT5
ZNF107	ZNF160	ZNF182	ZNF195	ZNF215	ZNF250	ZNF331
ZNF131	ZNF18	ZNF185	ZNF197	ZNF248	ZNF300	ZNF33
ZNF335	ZNF34	ZNF385B	ZNF398	ZNF484	ZNF521	ZNF570
ZNF33A	ZNF346	ZNF385D	ZNF442	ZNF518A	ZNF567	ZNF596
ZNF674	ZNF782	ZNF841	ZPBP	ZSCAN1	ZSCAN31	
ZNF687	ZNF839	ZP3	ZPBP2	ZSCAN18	ZSWIM2	
